# Gut Microbiota Modulation by Lysozyme as a Key Regulator of Vascular Inflammatory Aging

**DOI:** 10.34133/research.0704

**Published:** 2025-05-23

**Authors:** Chenyang Zhang, Xin Zhao, Hang Zhang, Tongtong Wang, Zhenyu Zhang, Yilin Yin, Hui Wang, Xiao Tong, Yuzheng Xue, Yahong Zhou, Fenglai Yuan, Xiuwu Bian, Hong Wei, Yuan Huang, Tianhao Liu

**Affiliations:** ^1^ Institute of Integrated Traditional Chinese and Western Medicine, Affiliated Hospital of Jiangnan University, Wuxi 214122, China.; ^2^ Wuxi School of Medicine, Jiangnan University, Wuxi 214122, China.; ^3^ Department of Rehabilitation Treatment, Jiangsu Rongjun Hospital, Wuxi 214062, China.; ^4^ Department of Pathology, Army Medical University, Chongqing 400038, China.; ^5^ Yu-Yue Pathology Scientific Research Center, Jinfeng Laboratory, Chongqing 401329, China.; ^6^ Department of Pediatrics, Affiliated Hospital of Jiangnan University, Wuxi 214122, China.; ^7^ Department of Gastroenterology, Affiliated Hospital of Jiangnan University, Wuxi 214122, Jiangsu, China.; ^8^ Wuxi Hospital Affiliated to Nanjing University of Chinese Medicine, Wuxi 214071, Jiangsu, China.; ^9^ National Center for Cardiovascular Diseases, Fuwai Hospital, Chinese Academy of Medical Sciences, Peking Union Medical College, Beijing 100037, China.

## Abstract

Vascular inflammatory aging is strongly associated with multimorbidity, including immunosenescence. Here, bioinformatic analysis indicated elevated expression of the lysozyme (LYZ) gene in age-dependent vascular diseases. *Lyz1* deficiency led to vascular inflammatory aging, including damage to indicators related to oxidative stress, vascular function, and inflammation in the serum and vascular tissues of wild-type (WT) and *Lyz1^−/−^* mice. The 16S ribosomal RNA sequencing of intestinal contents revealed increased *Bifidobacterium* and its metabolism of acetate, butyrate, omega-muricholic acid, propionate, and valeric acid in *Lyz1^−/−^* mice compared with that in WT mice. Additionally, RNA sequencing of vascular tissues identified differentially expressed genes in *Lyz1^−/−^* mice compared with those in WT mice, as well as enrichment of the common phosphatidylinositol 3-kinase (PI3K)–Akt signaling pathway. Vascular inflammatory aging phenotypes were detected in the blood vessels of antibiotic-treated and germ-free mice, and the PI3K–Akt signaling pathway was inhibited. Importantly, intravenous LYZ administration worsened the pathological conditions, whereas oral LYZ administration successfully restored the gut microbial balance and reversed the vascular inflammatory aging phenotypes. Collectively, this study establishes LYZ as a novel biomarker for age-related vascular diseases and the gut microbiota–PI3K–Akt axis as a promising therapeutic target.

## Introduction

Vascular aging plays a central role in morbidity and mortality among older adults with cardiovascular diseases [1,2]. “Aging” typically refers to chronological aging, which is suboptimal for estimating inflammation-led vascular aging compared with biological vascular aging. The latter may be beneficial for screening cardiovascular diseases at an early stage [3]. In the vascular system, oxidative stress, mitochondrial dysfunction, chronic low-grade inflammation, genomic instability, epigenetic changes, loss of protein homeostasis, and stem cell failure may contribute to structural and functional changes that accelerate biological vascular aging [4]. Aging vascular endothelial and smooth muscle cells exhibit a pro-inflammatory shift in gene expression, inducing the release of inflammatory cytokines (e.g., interleukin-6 and interleukin-1-beta [IL-1β]), tumor necrosis factor-alpha (TNF-α), adhesion molecules, and other pro-inflammatory mediators [5–9]. Cytokines and chemokines secreted from the endothelium induce damaging signals in vascular cells, nonvascular tissues, and organs. Consequently, endothelial senescence contributes to various cardiovascular and metabolic diseases [10,11].

Among cardiovascular diseases, inflammation has long been considered a key factor for atherosclerosis. Inflammatory aging is an important concept that can aid in understanding the complex interplay between chronic inflammation and aging. The inflammatory aging clock is strongly associated with multimorbidity, immunosenescence, frailty, cardiovascular aging, and longevity, making it an excellent indicator for tracking multiple diseases. Additional research has also identified significant contributors to inflammatory aging and inflammatory aging clock-related genes [12,13].

Gut dysbiosis is linked to longevity, health, and diseases and is therefore regarded as a potential biological aging estimator [14–16]. The concept of the “microbiota–inflammation–disease axis” has been explored in various diseases such as cancer, neurodegenerative disease, and diabetes, with focus on elucidating the relationship between gut dysbiosis, inflammatory bowel disease, and the role of inflammation in eliciting pathogenesis in other organs or systems [17–20]. Lysozyme (LYZ) plays an important role in bacterial lysis, by hydrolyzing the glycosidic bond between *N*-acetylmuramic acid and *N*-acetylglucosamine and destroying the peptidoglycan (PG)-rich cell walls of gram-positive bacteria [21]. The C-type LYZ is predominantly expressed in chordates and different classes of Arthropoda [21]. LYZ C-1, also known as 1,4-β-*N*-acetylmuramidase C or LYZ C-type P, is encoded by the lysozyme 1 gene (*Lyz1*), which is orthologous to human LYZ and is involved in the defense response to gram-positive and gram-negative bacteria. It has the highest expression in adult large (RPKM 3054.3) and small intestines (RPKM 742.7). LYZ can be secreted by the epithelium, neutrophils, or macrophages and subsequently delivered to bacterium-containing phagosomes. The epithelium secretes LYZ to kill bacteria at the site of infection and facilitates the release of pathogen-associated molecular patterns, including monomeric PG [22]. Extracellular insoluble PG elicits potent phagocyte chemotaxis via the complement pathway. LYZ activates pro-inflammatory immune responses in multiple ways, and failure to clear PG by LYZ can drive increased inflammation. Intestinal inflammation reportedly correlates with LYZ P deficiency [23], with LYZ mutation causing gastrointestinal, renal, and hepatic symptoms [24]. LYZ administration in mice with dextran sodium sulfate-induced colitis can alleviate intestinal inflammation [25]. LYZ also drives an anti-inflammatory response to alleviate inflammatory-driven pathology and limit inflammation [26,27].

Given that the gut microbiota is closely related to vascular inflammatory aging and LYZ is a key regulatory factor of gut microbiota, we hypothesized that the interaction between LYZ and gut microbiota mediates vascular inflammatory aging. Therefore, in the present study, we aimed to explore the roles of *Lyz1* and gut microbiota in vascular inflammatory aging using antibiotic-treated (ATBx) and germ-free (GF) *Lyz1^−/−^* mice and compared the findings with those in wild-type (WT) mice. Additionally, we probed LYZ changes in physiological aging by constructing models of aged mice and aged mice subjected to intragastric LYZ treatment and compared the findings with those in young WT mice. This study established LYZ as a novel biomarker for age-related vascular diseases and the gut microbiota–phosphatidylinositol 3-kinase (PI3K)–Akt axis as a promising therapeutic target.

## Results

### Age-dependent vascular pathogenesis involves elevated *LYZ* expression

**Fig. 1.**
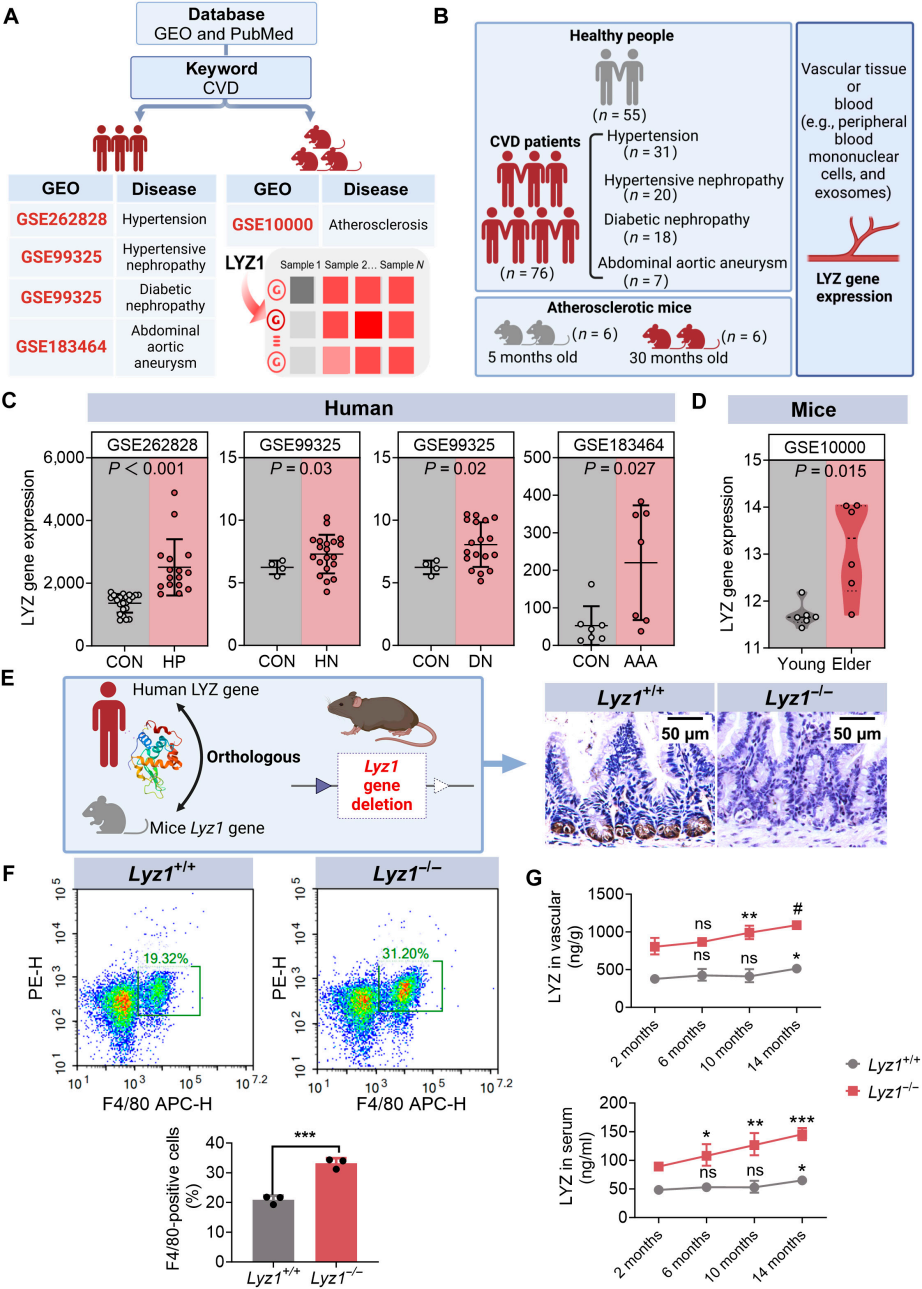
Elevated *LYZ* expression in age-related vascular diseases and construction of disease model mice. (A) Flowchart illustrating the data analysis process for the included studies. (B) Sample distribution and grouping scheme for the analyzed cohorts. (C) *LYZ* expression profiles of clinical populations with age-dependent diseases. CON, healthy controls; HP, patients with hypertension; HN, patients with hypertensive nephropathy; DN, patients with diabetic nephropathy; AAA, patients with hypertension abdominal aortic aneurysm. (D) *Lyz* expression pattern in mouse models of atherosclerosis. Young: 5-month-old mice; older: 30-month-old mice. (E) Generation and phenotypic validation of human *LYZ* homologous gene knockout mice. Left: Schematic diagram of mouse model construction. Right: Immunohistochemical staining of lysozyme (LYZ) in ileum tissue. (F) Flow cytometry analysis of F4/80+ cell populations in mouse peripheral blood. ***P < 0.001. (G) Age-dependent *Lyz* expression in the vascular tissues and serum of *Lyz1^+/+^* and *Lyz1^−/−^* mice. *P < 0.05, **P < 0.01, and ***P < 0.001 compared to 2-month-old mice; ns, not significant. ^#^*P* < 0.05 compared to the preceding age group. *Lyz1*^*+/*+^, wild-type mice; *Lyz1^−/−^*, Lyz1 gene knockout mice. GEO, Gene Expression Omnibus; CVD, cardiovascular disease; PE-H, phycoerythrin height of electric pulse signal; APC-H, allophycocyanin height of electric pulse signal.

A comprehensive search of the Gene Expression Omnibus and PubMed databases conducted up to November 2024 using the keyword “cardiovascular disease” identified 8 datasets, including 3 clinical studies and 4 laboratory studies (Fig. 1A and Table S1). The clinical studies analyzed vascular tissues or intravascular materials from 55 healthy individuals and 76 patients with vascular-related diseases, including hypertension (*n* = 31), hypertensive nephropathy (*n* = 20), diabetic nephropathy (*n* = 18), and abdominal aortic aneurysm (*n* = 7). These studies revealed elevated *LYZ* expression in diseased tissues (Fig. 1B and C). Laboratory studies further showed increased LYZ expression in the vascular tissues of aged atherosclerotic mice (Fig. 1B and D). These findings suggest that LYZ is a potential biomarker for age-dependent vascular diseases.

As a nonspecific antimicrobial peptide, LYZ plays a protective role in maintaining intestinal homeostasis, and its absence disrupts intestinal barrier integrity, exacerbating the progression of age-dependent vascular diseases. We investigated the role of LYZ deficiency in these diseases by generating *Lyz1* knockout mice (*Lyz1^−/−^*), characterized by the deletion of Paneth cells in intestinal crypts and age-dependent increases in LYZ levels in vascular tissues (Fig. 1E and G). Flow cytometry indicated that the loss of Paneth cell-derived LYZ led to a compensatory increase in macrophage-derived LYZ in vascular tissues (Fig. 1F).

### *Lyz1* deficiency-induced vascular inflammatory aging is associated with the gut microbiota

**Fig. 2.**
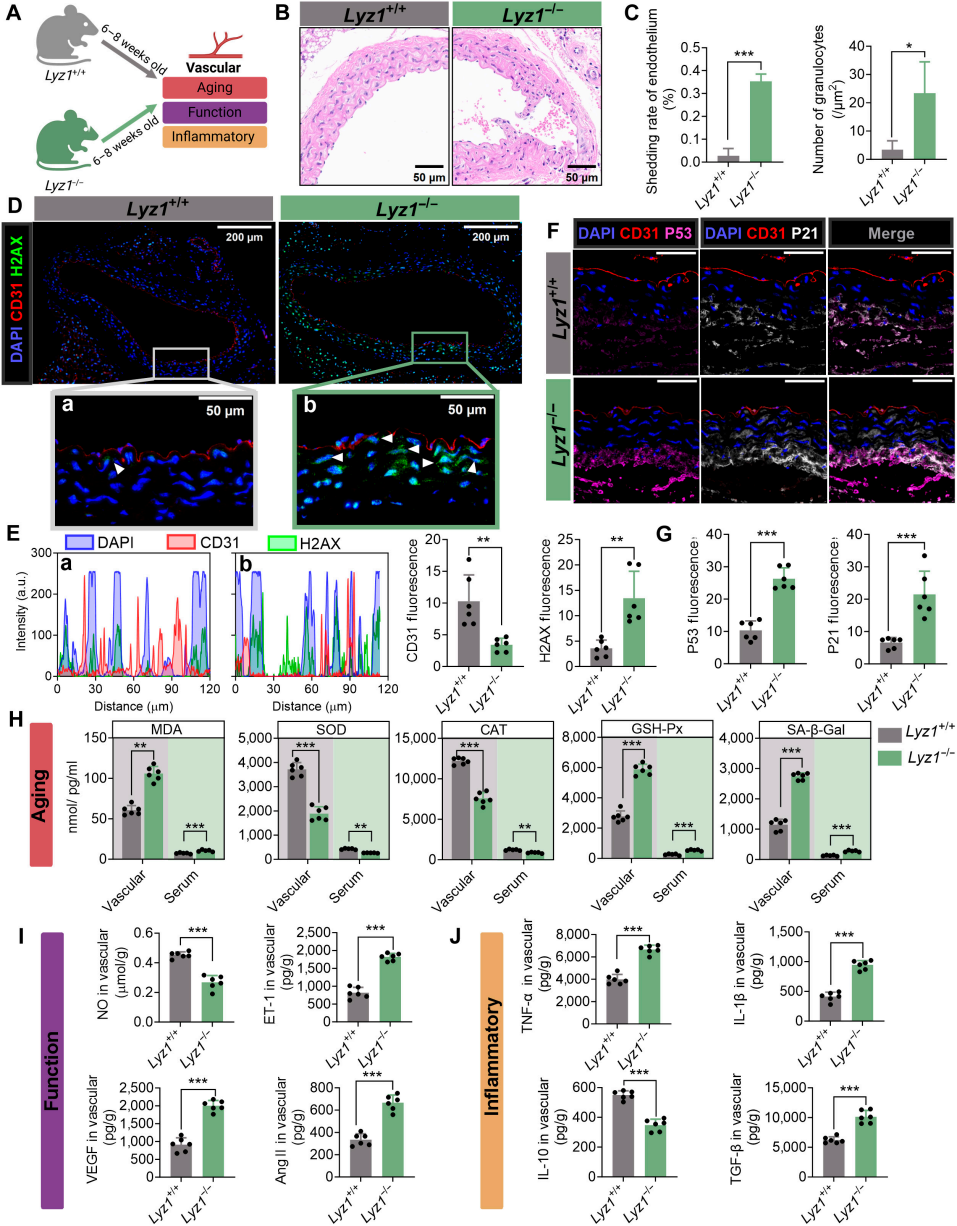
*Lyz1* deficiency induces vascular aging, functional impairment, and inflammatory responses in mice. (A) Experimental design and group allocation schematic. (B) Representative hematoxylin and eosin (HE) staining of aortic sections. (C) Assessment of endothelial cell detachment rates and vascular granulocyte counts. (D) Immunofluorescence analysis of aortic vessels. Nuclei are stained with 4′,6-diamidino-2-phenylindole (DAPI; blue), CD31 (red; endothelial cell marker), and H2A histone family member X (H2AX; green; DNA damage marker). (E) Quantification of nuclear colocalization and fluorescence intensity of CD31 and H2AX. (F) Immunofluorescence staining of aortic vessels showing nuclei (DAPI; blue), CD31 (red), p53 (purple), and p21 (white). Scale bar: 50 μm. (G) Quantitative analysis of p53 and p21 fluorescence intensity. (H) Alterations in vascular aging markers, including malondialdehyde (MDA), superoxide dismutase (SOD), catalase (CAT), glutathione peroxidase (GSH-Px), and senescence-associated β-galactosidase (SA-β-Gal). (I) Changes in vascular functional markers, including nitric oxide (NO), endothelin-1 (ET-1), vascular endothelial growth factor (VEGF), and angiotensin II (Ang II). (J) Modulation of inflammatory markers, including tumor necrosis factor-α (TNF-α), interleukin-1β (IL-1β), interleukin-10 (IL-10), and transforming growth factor-β (TGF-β). Data are presented as mean ± standard error of the mean (SEM). Statistical significance was determined using analysis of variance (ANOVA) or a Student *t* test: *P < 0.05; **P < 0.01; ***P < 0.001; ns, not significant. *Lyz1^+/+^*, wild-type mice; *Lyz1^−/−^*, Lyz1 knockout mice.

We examined markers related to vascular aging, function, and inflammation and found that *Lyz1* deficiency induced vascular aging (Fig. 2A). Compared with *Lyz1^+/+^* mice, *Lyz1^−/−^* mice displayed endothelial cell shrinkage, detachment, and disorganized arrangement; partial detachment of the internal elastic membrane; reduced curvature of the medial elastic structure; localized intimal thickening with fibrosis; and scattered granulocytes with deeply stained nuclei in the adventitia (Fig. 2B). Endothelial cell detachment rates and granulocyte counts were also significantly increased (Fig. 2C). DNA damage, a hallmark of aging, can be confirmed by assessing H2A histone family member X (H2AX) phosphorylation, which indicates DNA damage and initiation of DNA repair mechanisms [28,29]. *Lyz1^−/−^* mice exhibited enhanced H2AX fluorescence intensity in nuclei, indicating increased DNA damage (Fig. 2D and E). Additionally, the expression of the cell cycle regulatory proteins p21 and p53, which are markers of cellular senescence, was significantly elevated in the vascular media and adventitia of *Lyz1^−/−^* mice (Fig. 2F and G). Vascular tissues and serum from *Lyz1^−/−^* mice also showed increased levels of the aging marker senescence-associated β-galactosidase (SA-β-Gal) and modulation of oxidative stress factors (elevated malondialdehyde [MDA] and reduced superoxide dismutase [SOD], catalase, and glutathione peroxidase levels) (Fig. 2H), suggesting that *Lyz1* knockout promotes vascular aging.

Endothelial function-related markers, including the vasoprotective molecule nitric oxide (NO), the vasoconstrictors endothelin-1 (ET-1) and angiotensin II (Ang II), and the vascular permeability regulator vascular endothelial growth factor (VEGF) [10,11], were altered in *Lyz1^−/−^* mice, indicating vascular dysfunction, with NO levels being significantly decreased and ET-1, VEGF, and Ang II levels being significantly increased (Fig. 2I). Furthermore, the levels of the macrophage-related inflammatory cytokines TNF-α, IL-1β, and tumor growth factor-beta (TGF-β) were elevated, whereas interleukin-10 (IL-10) levels were reduced in *Lyz1^−/−^* mice compared with those in in *Lyz1^+/+^* mice (Fig. 2J). These findings suggest that Lyz1 deficiency may contribute to macrophage-associated inflammation and vascular aging.

**Fig. 3.**
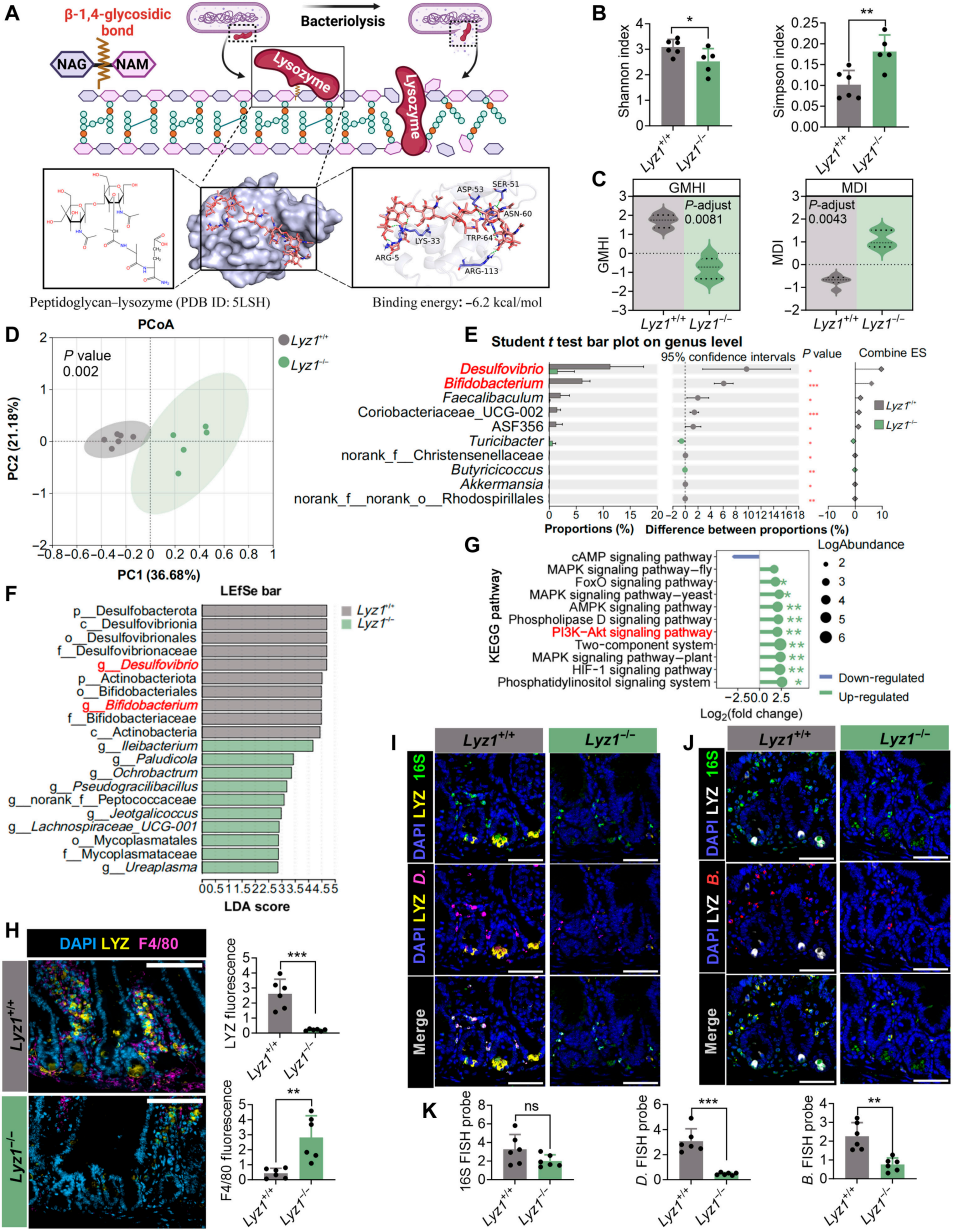
Alterations in the gut microbiota and macrophage recruitment in *Lyz1^−/−^* mice. (A) Schematic representation of LYZ-mediated cleavage of β-1,4-glycosidic bonds in bacterial peptidoglycan. NAG, *N*-acetyl-d-(+)-glucosamine; NAM, *N*-acetylmuramic acid; SER, serine; ASP, aspartic acid; ASN, asparagine; TRP, tryptophan; LYS, lysine; ARG, arginine. (B) Differences in α diversity assessed by Shannon and Simpson indices. (C) Changes in gut microbiota health and diversity evaluated using the gut microbiome health index (GMHI) and microbiome dysbiosis index (MDI). (D) Principal coordinate analysis (PCoA) based on operational taxonomic unit (OTU) abundance, illustrating β diversity between groups. (E) Top 10 differentially abundant bacterial genera identified by *t* test. (F) Linear discriminant analysis (LDA) showing the top 10 most abundant bacterial taxa in the gut microbiota. (G) Kyoto Encyclopedia of Genes and Genomes (KEGG) pathway enrichment analysis revealing the top 11 significantly altered signaling pathways. (H) Immunofluorescence colocalization of LYZ and macrophage marker F4/80 in intestinal tissues. Nuclei are stained with DAPI (blue), LYZ is shown in yellow, and F4/80 is shown in in purple. Scale bars: 30 μm (×40) and 20 μm (×65). Bar graphs quantify differences in LYZ and F4/80 fluorescence intensity. (I) Fluorescence in situ hybridization (FISH) showing colocalization of LYZ and *Desulfovibrio*. Nuclei are stained with DAPI (blue), total bacteria are labeled with 16S (green), LYZ is shown in yellow, and *Desulfovibrio* is shown in in purple. Scale bars: 30 μm (×40) and 20 μm (×65). (J) FISH analysis of LYZ and *Bifidobacterium* colocalization. Nuclei are stained with DAPI (blue), total bacteria are labeled with 16S (green), LYZ is shown in white, and *Bifidobacterium* is shown in red. Scale bars: 30 μm (×40) and 20 μm (×65). (K) Quantification of fluorescence intensity for colocalization of LYZ with total bacteria, *Desulfovibrio*, and *Bifidobacterium*. Data are presented as mean ± SEM. Statistical significance was determined using ANOVA or a Student *t* test: *P < 0.05; **P < 0.01; ***P < 0.001; ns, not significant. *Lyz1^+/+^*, wild-type mice; *Lyz1^−/−^*, Lyz1 knockout mice. LEfSe, linear discriminant analysis effect size; cAMP, cyclic adenosine monophosphate; MAPKs, mitogen-activated protein kinases; AMPK, adenosine 5′-monophosphate (AMP)-activated protein kinase; HIF-1, hypoxia inducible factor-1.

Our results showed that LYZ exerts its antimicrobial effects by disrupting the β-1,4-glycosidic bonds present in bacterial cell walls (Fig. 3A). Gut microbiota sequencing revealed significant differences in *Lyz1^−/−^* mice, including changes in α-diversity and distinct β-diversity clustering (Fig. 3B to D and Fig. S1A). The composition of dominant bacterial species varied across taxonomic levels (Fig. S1B to E). Differential abundance and linear discriminant analysis effect size analyses revealed a significant reduction in *Bifidobacterium* and *Desulfovibrio* abundance in *Lyz1^−/−^* mice (Fig. S1B and C and Fig. 3E and F). Fluorescence in situ hybridization (FISH) revealed that the loss of LYZ in Paneth cells led to the reduced colonization of *Desulfovibrio* and *Bifidobacterium* in intestinal crypts (Fig. 3I to K). Functional analysis of the gut microbiota suggested that *Lyz1* deficiency might affect microbial pathways related to mitogen-activated protein kinase, Foxo, and PI3K–Akt signaling (Fig. 3E). Immunofluorescence colocalization revealed that *Lyz1* deficiency activated intestinal macrophages and enhanced their recruitment (Fig. 3H).

### *Lyz1* deficiency-mediated vascular inflammatory aging is related to *Bifidobacterium* and its metabolites

**Fig. 4.**
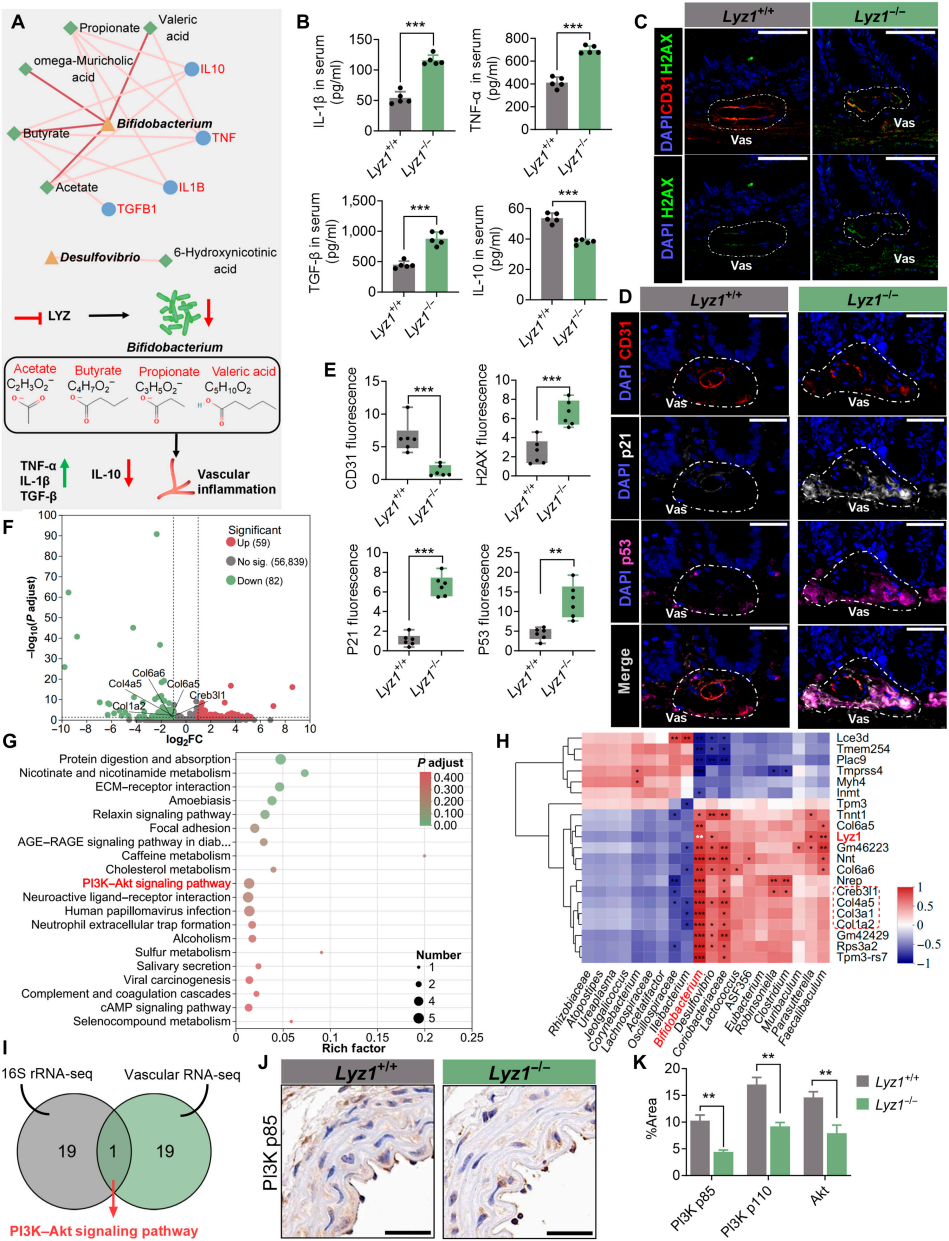
Mechanism of Lyz1 deficiency-mediated vascular inflammation and aging driven by *Bifidobacterium*. (A) Metabolic and gene network analysis of *Bifidobacterium* and *Desulfovibrio*. (B) Changes in serum inflammatory markers, including TNF-α, IL-1β, TGF-β, and IL-10. (C) Immunofluorescence staining of intestinal vasculature. Nuclei are stained with DAPI (blue), CD31 (red; endothelial cell marker), and γH2AX (green; DNA damage marker). Scale bars: 200 and 50 μm. (D) Immunofluorescence staining of intestinal vasculature. Nuclei are stained with DAPI (blue), CD31 (red), p53 (purple), and p21 (white). Scale bars: 200 and 50 μm. (E) Quantification of fluorescence intensity for CD31, H2AX, p53, and p21. (F) Differential gene expression in the vascular tissues of *Lyz1^−/−^* and *Lyz1^+/+^* mice, identifying 141 differentially expressed genes (59 up-regulated, 82 down-regulated). (G) KEGG pathway enrichment analysis showing altered signaling pathways in vascular tissues. (H) Correlation analysis between vascular tissue differentially expressed genes and bacterial abundance. (I) Overlap of KEGG pathway enrichment results from 16S sequencing and vascular transcriptome analysis. (J) Immunohistochemical staining of PI3K (p85) in vascular tissues of *Lyz1^−/−^* mice. Scale bars: 30 and 20 μm. (K) Quantitative analysis of PI3K (p85), PI3K (p110), and AKT expression in vascular tissues. PI3K, phosphatidylinositol 3-kinase; AKT, protein kinase B. Data are presented as mean ± SEM. Statistical significance was determined using ANOVA or a Student *t* test: *P < 0.05; **P < 0.01; ***P < 0.001; ns, not significant. *Lyz1^+/+^*, wild-type mice; *Lyz1^−/−^*, Lyz1 knockout mice.

To simulate the altered metabolic environment of *Lyz1^−/−^* mice, we modeled metabolites from the top 20 bacterial species, resulting in the identification of 11 bacteria and 271 vascular inflammation-related genes. Among them, 10 bacteria, including *Bifidobacterium*, *Coriobacteriaceae*, and *Butyricicoccus*, interacted with 58 host genes through 17 metabolites to promote vascular inflammation (Figs. S2 and S3). Notably, *Bifidobacterium* played a central role by influencing the metabolism of acetate, butyrate, omega-muricholic acid, propionate, and valeric acid and up-regulating macrophage-related inflammatory factors (TNF-α, IL-1β, and TGF-β) while down-regulating IL-10 (Fig. 4A and B). These changes contributed to vascular dysfunction (Fig. S1E and F). *Lyz1^−/−^* mice exhibited intestinal barrier damage, shortened villi, reduced crypt height, and altered goblet cell distribution (Fig. S4A to E), as well as vascular endothelial cell cycle arrest and DNA damage (Fig. 4C to E), linking systemic vascular aging to intestinal vascular aging.

Transcriptomic analysis of vascular tissues revealed 141 differentially expressed genes in *Lyz1^−/−^* mice compared with those in *Lyz1^+/+^* mice (Fig. 4F). Pathway enrichment analysis revealed the down-regulation of PI3K–Akt signaling in *Lyz1^−/−^* vascular tissues (Fig. 4F and G and Fig. S4D), with genes primarily involved in the secretion of proteins related to cell membranes and organelles and involved in cellular processes, biological regulation, and developmental processes related to the circulatory and cardiovascular systems (Fig. S5A to D). Key aging-related genes such as *Col6a6*, *Col6a5*, *Col4a5*, and *Col1a2* were significantly down-regulated in *Lyz1^−/−^* mice and associated with *Bifidobacterium* abundance (Fig. 4H). The PI3K–Akt pathway was the only common pathway enriched in both gut microbiota and vascular gene analyses (Fig. 4I). Immunohistochemistry confirmed the reduced expression of PI3K (p110), PI3K (p85), and Akt in *Lyz1^−/−^* mice (Fig. 4J and K and Fig. S4F and G). These findings indicate that a potential link between reduced *Bifidobacterium* abundance, altered metabolite production, and PI3K–Akt signaling plays a significant role in vascular inflammatory aging.

### Gut microbiota depletion mediates LYZ-induced vascular inflammatory aging in Paneth cells

**Fig. 5.**
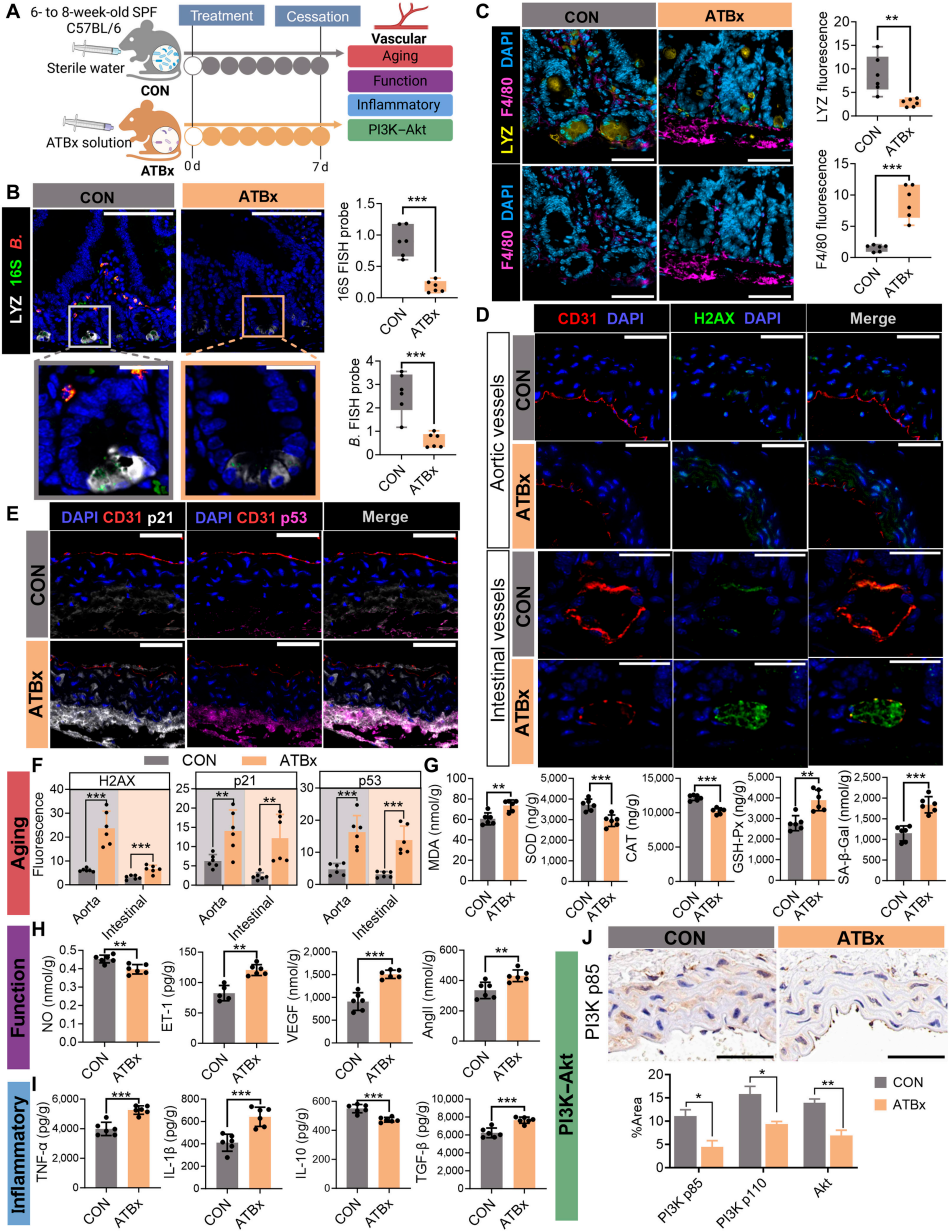
Mechanistic role of gut microbiota in Paneth cell LYZ-mediated vascular inflammation and aging revealed by ATBx mouse models. (A) Schematic of experimental design and key analytical endpoints. (B) FISH showing colocalization of LYZ and *Bifidobacterium* in intestinal tissues. LYZ is labeled in white, total bacteria with 16S in green, and *Bifidobacterium* in red. Bar graphs quantify the fluorescence intensity of LYZ colocalization with total bacteria and *Bifidobacterium*. Scale bars: 30 μm (×40) and 20 μm (×65). (C) Immunofluorescence colocalization of LYZ and macrophage marker F4/80. Nuclei are stained with DAPI (blue), LYZ in yellow, and F4/80 in purple. Scale bars: 30 μm (×40) and 20 μm (×65). Bar graphs compare LYZ and F4/80 fluorescence intensity between groups. (D) Immunofluorescence staining of aortic and intestinal vasculature. Nuclei are stained with DAPI (blue), CD31 (red; endothelial marker), and H2AX (green; DNA damage marker). Scale bars: 200 μm (overview) and 50 μm (magnified). (E) Immunofluorescence staining of aortic vessels. Nuclei are stained with DAPI (blue), CD31 (red), p53 (purple), and p21 (white). Scale bars: 200 μm (overview) and 50 μm (magnified). (F) Quantification of H2AX, p21, and p53 fluorescence intensity in aortic and intestinal vasculature. H2AX: DNA damage marker. (G) Alterations in vascular aging markers: MDA, SOD, CAT, GSH-Px, and SA-β-Gal. (H) Changes in vascular functional markers: NO, ET-1, VEGF, and Ang II. (I) Modulation of inflammatory markers: TNF-α, IL-1β, IL-10, and TGF-β. (J) Immunohistochemical staining and quantitative analysis of PI3K (p85), PI3K (p110), and AKT in intestinal tissues. Scale bars: 30 μm (overview) and 20 μm (magnified). Data are presented as mean ± SEM. Statistical significance: *P < 0.05; **P < 0.01; ***P < 0.001; ns, not significant. SPF, specific pathogen-free; CON, control group; ATBx, antibiotic-treated mice.

**Fig. 6.**
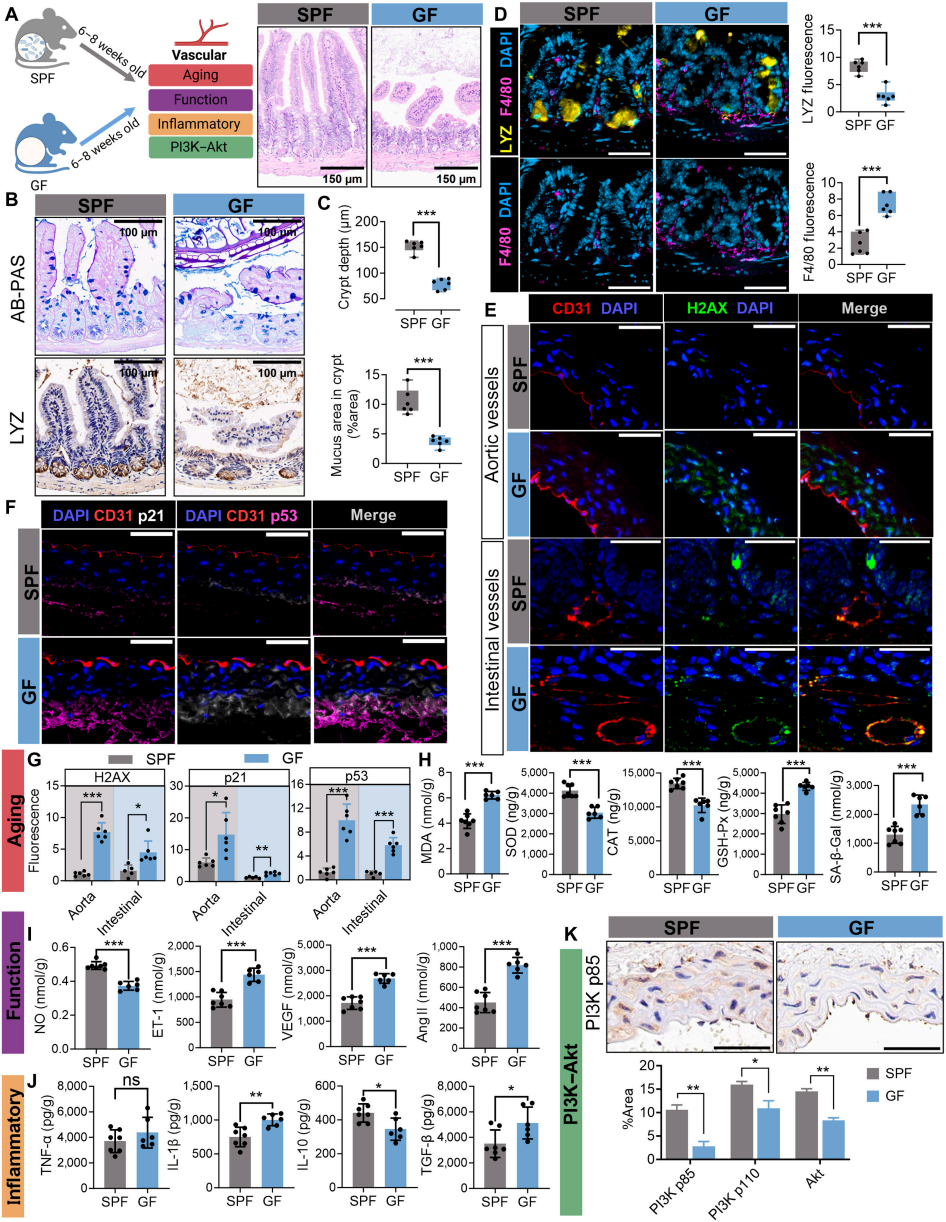
Gut microbiota mediates Paneth cell LYZ-driven vascular inflammation and aging in germ-free (GF) mouse models. (A) Experimental design, key endpoints, and intestinal morphology. (B) Alcian Blue–periodic acid–Schiff (AB-PAS) staining of intestinal sections and LYZ expression. (C) Quantification of crypt depth and mucus area in intestinal crypts. (D) Immunofluorescence colocalization of LYZ and macrophage marker F4/80. Nuclei are stained with DAPI (blue), LYZ in yellow, and F4/80 in purple. Scale bars: 30 μm (×40) and 20 μm (×65). Bar graphs compare LYZ and F4/80 fluorescence intensity. (E) Immunofluorescence staining of aortic and intestinal vasculature. Nuclei are stained with DAPI (blue), CD31 (red; endothelial marker), and H2AX (green; DNA damage marker). Scale bars: 200 μm (overview) and 50 μm (magnified). (F) Immunofluorescence staining of aortic vessels. Nuclei are stained with DAPI (blue), CD31 (red), p53 (purple), and p21 (white). Scale bars: 200 μm (overview) and 50 μm (magnified). (G) Quantification of H2AX, p21, and p53 fluorescence intensity in aortic and intestinal vasculature. H2AX, DNA damage marker. (H) Changes in vascular aging markers: MDA, SOD, CAT, GSH-Px, and SA-β-Gal. (I) Alterations in vascular functional markers: NO, ET-1, VEGF, and Ang II. (J) Modulation of inflammatory markers: TNF-α, IL-1β, IL-10, and TGF-β. (K) Immunohistochemical staining and quantitative analysis of PI3K (p85), PI3K (p110), and AKT in intestinal tissues. Data are presented as mean ± SEM. Statistical significance: *P < 0.05; **P < 0.01; ***P < 0.001; ns, not significant. CON, control group; GF, germ-free mice.

We established ATBx and GF mouse models to elucidate the interplay between gut microbiota and LYZ in vascular inflammatory aging. Given the inherent immunological heterogeneity of these models [30], this study implemented a dual-model complementary validation strategy to exclude confounding effects arising from immune developmental disparities, thereby ensuring mechanistic specificity in delineating gut microbiota-mediated pathways underlying vascular senescence (Figs. 5 and 6A). Compared with the control (CON) mice, the ATBx mice exhibited endothelial cell shrinkage, detachment, and disorganized arrangement in aortic vessels; incomplete intimal structure; and increased granulocyte counts (Fig. S6A and B). While crypt depth and villus length remained unchanged in the ATBx mice (Fig. S6A and C), goblet cell density was significantly reduced, and mucosal edema was detected (Fig. S6A and D). FISH revealed a significant reduction in total bacterial counts and *Bifidobacterium* colonization in the ATBx mice, accompanied by low LYZ expression in Paneth cells and increased macrophage recruitment and activation (Fig. 5B and C). The vascular aging markers H2AX, p53, and p21 were elevated in both the aortic and intestinal vessels of ATBx mice, along with the disruption of CD31 expression and endothelial cell integrity (Fig. 5D to F and Fig. S6E). ATBx mice also exhibited increased MDA and SA-β-Gal levels, decreased SOD and catalase levels, and altered levels of vascular function markers (NO, ET-1, VEGF, and Ang II) and inflammatory cytokines (TNF-α, IL-1β, TGF-β, and IL-10) in vascular tissues and serum (Fig. 5G to I and Fig. S6F to H). Additionally, the expression of PI3K (p110), PI3K (p85), and Akt was significantly reduced in ATBx mice, indicating PI3K–Akt pathway suppression (Fig. 5J and Fig. S6I and J).

To further understand the impact of gut microbiota, we conducted validation experiments using GF mice. GF mice exhibited intestinal mucosal damage, crypt collapse, reduced crypt depth, a decreased mucus area, and low LYZ expression (Fig. 6A to C). Similar to ATBx mice, GF mice showed increased expression of H2AX, p53, and p21 in vascular tissues, with elevated MDA and SA-β-Gal levels and alterations in vascular function and inflammatory markers (Fig. 6E to J and Fig. S7A to G). However, compared with ATBx mice, which showed only slight damage to the intestinal mucosal barrier, GF mice had significant changes in intestinal tissue morphology, which was manifested by a significant reduction in villus length and crypt depth (Fig 6A and B and Fig. S6A). The expression of PI3K (p110), PI3K (p85), and Akt was also reduced in GF mice, further supporting the involvement of the PI3K–Akt pathway in vascular inflammatory aging (Fig. 6K and Fig. S7H and J). Collectively, these results indicate that the gut microbiota regulates LYZ expression and contributes to vascular inflammatory aging, likely via the PI3K–Akt pathway.

### LYZ enhances *Bifidobacterium* colonization and alleviates vascular inflammatory aging

**Fig. 7.**
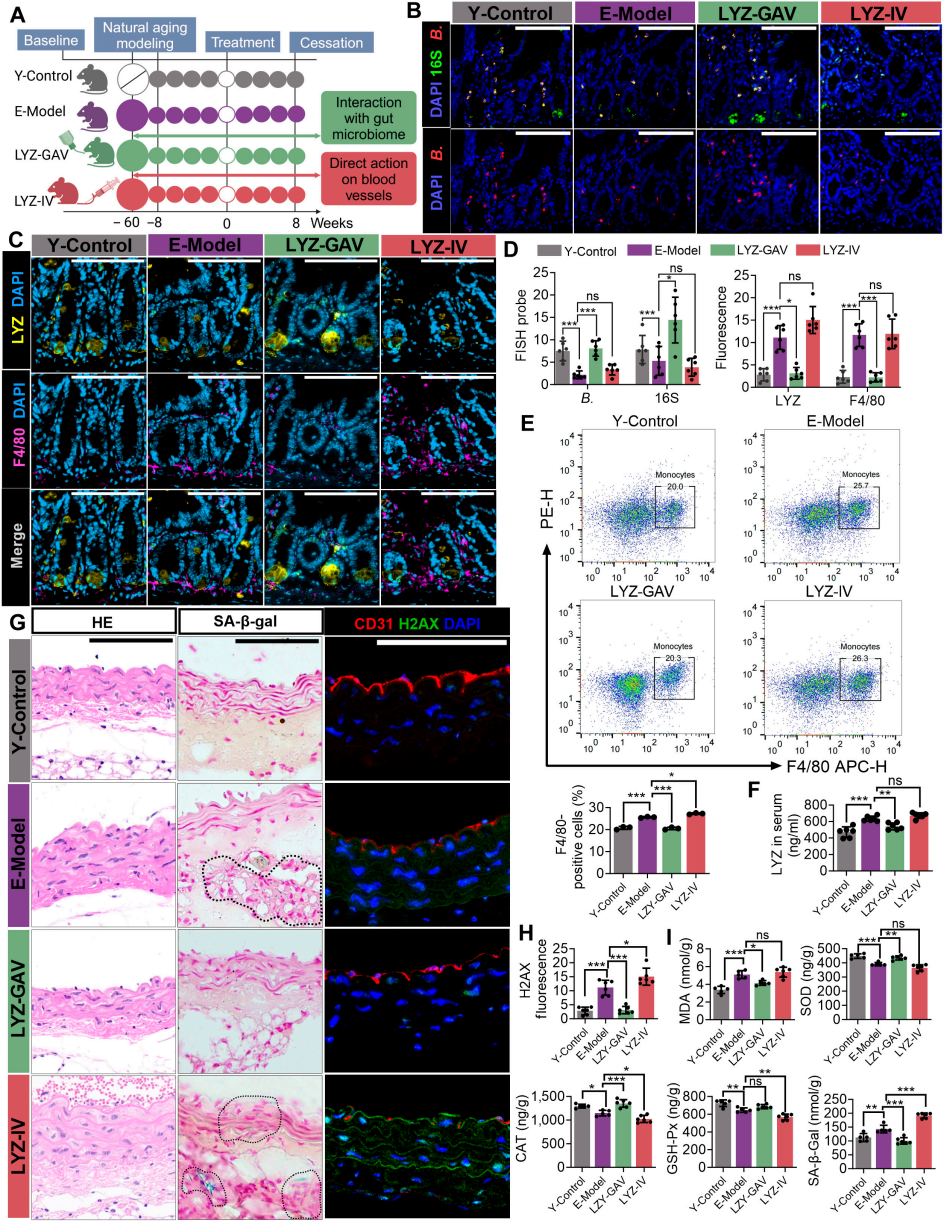
Oral LYZ supplementation alleviates vascular inflammation and aging in natural aging mice. (A) Schematic of experimental group allocation. (B) FISH showing localization of *Bifidobacterium* in intestinal tissues. Nuclei are stained with DAPI (blue), total bacteria with 16S (green), and *Bifidobacterium* in red. (C) Immunofluorescence colocalization of LYZ and macrophage marker F4/80. Nuclei are stained with DAPI (blue), LYZ in yellow, and F4/80 in purple. (D) Quantification of total bacteria and *Bifidobacterium* abundance, as well as fluorescence intensity of LYZ and F4/80 in intestinal tissues. (E) Flow cytometry analysis and quantification of F4/80+ cells in peripheral blood. (F) Quantification of serum LYZ levels. (G) Assessment of aortic aging markers. Left: HE staining of aortic vessels. Middle: SA-β-Gal expression in aortic vessels. Right: Immunofluorescence staining of aortic vessels. Nuclei are stained with DAPI (blue), CD31 (red; endothelial marker), and H2AX (green; DNA damage marker). (H) Quantification of H2AX in vascular tissues. γH2AX: DNA damage marker. (I) Changes in vascular aging markers: MDA, SOD, CAT, GSH-Px, and SA-β-Gal. Data are presented as mean ± SEM. Statistical significance: *P < 0.05; **P < 0.01; ***P < 0.001; ns, not significant. Y-Control, young control mice; E-Model, elderly model mice; LYZ-GAV, LYZ gavage; LYZ-IV, LYZ intravenous injection.

Large proteins such as LYZ cannot directly cross the intestinal barrier into the bloodstream but can interact with the gut microbiota as “prebiotics”. Thus, we explored the therapeutic potential of LYZ in alleviating vascular inflammatory aging by administering LYZ orally (LYZ-GAV) or intravenously (LYZ-IV) to aged (60-week-old) mice (E-Model) (Fig. 7A). Compared with young (8-week-old) control mice (Y-Control), the E-Model mice showed reduced total bacterial counts and *Bifidobacterium* colonization, which were partially restored by oral LYZ administration, but not by intravenous LYZ administration (Fig. 7B and D). Oral LYZ administration also restored the intestinal LYZ and macrophage levels, whereas intravenous LYZ administration had no effect or even exacerbated vascular aging (Fig. 7C and D). SA-β-Gal activity and H2AX expression, markers of DNA damage, were reduced in LYZ-GAV mice, but not in LYZ-IV mice (Fig. S8A to C). Vascular integrity and macrophage counts improved in LYZ-GAV mice, whereas no significant changes were identified in LYZ-IV mice (Fig. 7E to G). Additionally, serum and vascular tissue markers of oxidative stress and inflammation were ameliorated by oral LYZ administration, but not by intravenous LYZ administration (Fig. 7I and Fig. S8D to F). These findings suggest that intravenous LYZ administration exacerbates vascular aging, whereas oral administration of LYZ alleviates vascular inflammatory aging.

## Discussion

**Fig. 8.**
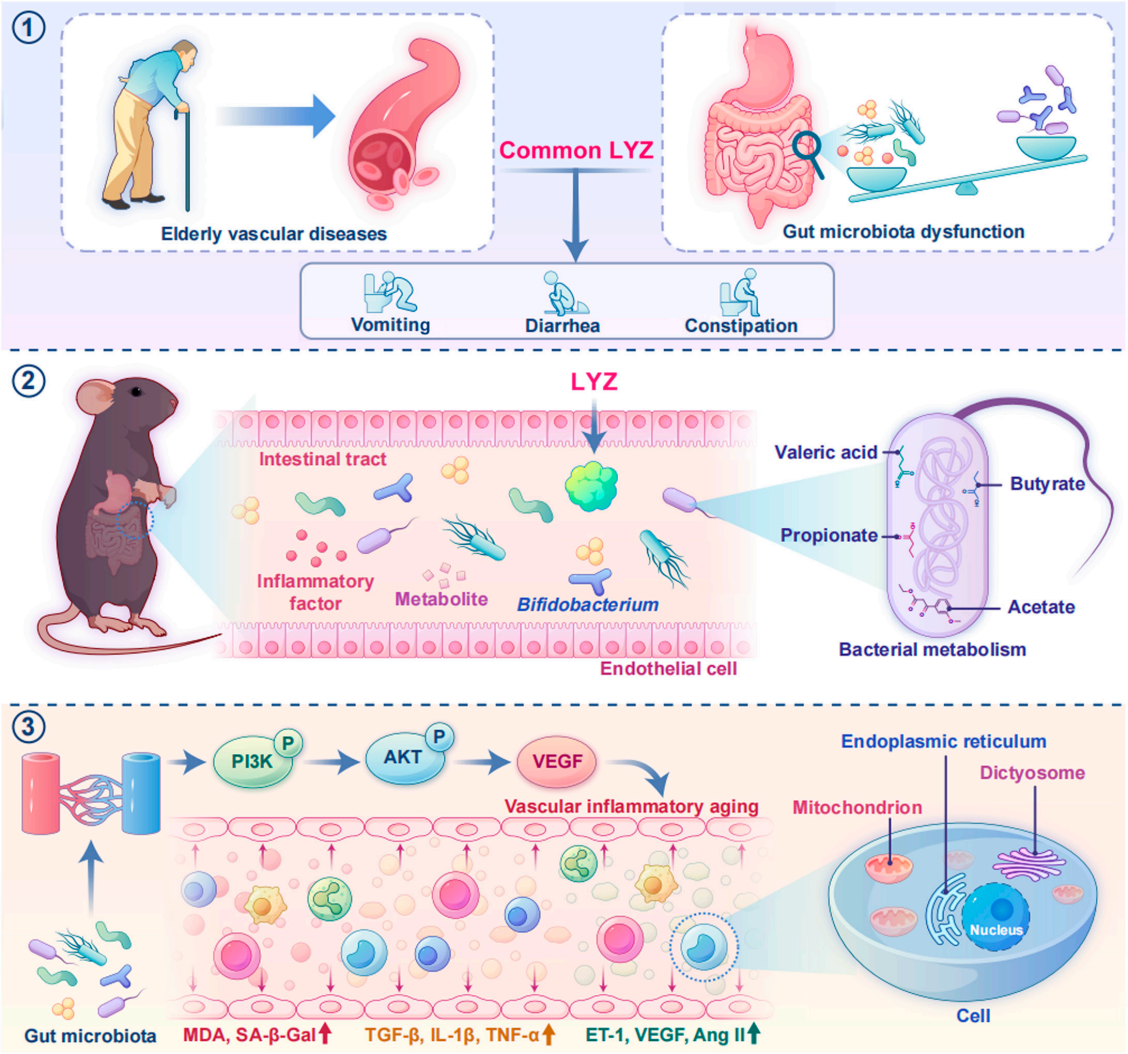
Schematic diagram depicting that the gut microbial response to LYZ alleviates vascular inflammation aging.

Our study revealed that the LYZ1 deficiency exacerbated morphological changes, aging, and functional decline in vascular endothelium. Furthermore, LYZ modulated the gut microbiota, especially *Bifidobacterium*. Our findings elucidated the potential molecular mechanism by which LYZ participates in vascular inflammatory aging via *Bifidobacterium* and its metabolites affecting the PI3K–Akt pathway. The ATBx model confirmed that gut microbiota regulates vascular inflammatory aging by modulating intestinal LYZ levels, which was further validated in GF mouse models. Additionally, vascular inflammatory aging was reversed by oral LYZ administration but was exacerbated by intravenous LYZ administration. Thus, our study reveals that LYZ is a key regulator of vascular inflammatory aging (Fig. 8).

Pathological processes of vascular diseases and inflammation-related aging were often accompanied by elevated plasma LYZ levels. LYZ is a plasma biomarker of atherosclerosis [31], and *LYZ* is associated with vascular-related diseases, including hypertension, diabetic nephropathy, and abdominal aortic aneurysm, with significantly elevated LYZ expression in peripheral blood and other tissues [32]. In our study, we detected *Lyz* expression in the vasculature of 5- and 30-month-old atherosclerotic mice. Age-segmented mice were often used to study aging models, which could simulate different degrees of aging [33,34]. Collectively, these results demonstrate that LYZ expression is significantly increased in aged vascular diseases, in both humans and mice. While the interaction between the gut microbiome and host genes was successful, expanding these investigations to other organs harboring diverse microbial populations is important [31,35].

LYZ has good bacterial lysis ability, especially against gram-positive bacteria owing to their PG-rich cell walls [21]. LYZ protects the intestine, and its abnormal expression can manifest in various disease states. Secretory autophagy redirects LYZ during bacterial infection. When the secretion pathway is blocked, the risk of Crohn’s disease increases [36]. LYZ messenger RNA was significantly up-regulated in the colon epithelial cells of patients with ulcerative colitis and Crohn’s disease [37]. This study also found that *Lyz1^−/−^* mice exhibited a disordered gut microbiota. Microbial analysis showed that the gut microbiota of *Lyz1^−/−^* mice underwent changes, characterized by the depletion of beneficial bacterial communities such as *Bifidobacterium*, which may exacerbate vasculitis and aging. Acetate, butyrate, propionate, valeric acid, and omega-muricholic acid are important short-chain fatty acids and bile acid metabolites produced by *Bifidobacterium* [38,39]. Their relationship with vascular inflammatory aging has recently received considerable attention as they play an important role in delaying vascular inflammatory aging via mechanisms such as anti-inflammatory, antioxidant, immune, and metabolic regulation [39–41].

The gut microbiota regulates signaling pathways such as nuclear factor-kappa B and PI3K–Akt, which affect the host’s inflammatory response [42,43]. The PI3K–Akt pathway is involved in cell proliferation, apoptosis, and metastasis. Activation of PI3K–Akt signaling, along with telomere shortening, impaired autophagy, mitochondrial dysfunction, immune aging, and low-grade chronic inflammation, is a common mechanism underlying multiple diseases. As the PI3K–Akt pathway is critical for cellular senescence, inhibiting this pathway may extend the lifespan [44,45].

Vascular endothelial cells are constantly exposed to numerous circulating signaling molecules, making them important targets for various pro- and anti-inflammatory cytokines [46–48]. The PI3K–Akt signaling pathway lays an important role in angiogenesis, with downstream factors of Akt, including endothelial NO synthase, VEGF, and mammalian rapamycin targets, being crucial for the growth, proliferation, and apoptosis of endothelial cells and cardiovascular homeostasis [49–51]. Notably, we found that the loss of LYZ in Paneth cells leads to a compensatory increase in LYZ in macrophages in blood vessels.

To the best of our knowledge, this is the first study to reveal that LYZ regulates vascular inflammatory aging through the gut microbiota–PI3K–Akt axis, breaking through the traditional perspective of “local vascular lesions” and proposing a new paradigm of “gut–vascular inflammatory aging axis”. While the study suggests a role for *Bifidobacterium* metabolites in modulating the PI3K–Akt pathway, the specific mechanisms remain unclear. *Bifidobacterium animalis* subsp. *lactis BI-04* strain can inhibit the apoptosis of colonic epithelial cells by up-regulating the PI3K–Akt pathway and up-regulating p53 gene expression [52]. Given the well-documented interactions between bacterial metabolites and signaling pathways, single-bacterium colonization experiments and metabolomic analyses should be performed in future studies to elucidate these mechanisms more precisely [53,54]. Our study also demonstrated that oral LYZ administration ameliorated vascular inflammatory aging by restoring *Bifidobacterium* colonization, whereas intravenous LYZ administration was ineffective or even detrimental, underscoring the critical role of the local gut microbial microenvironment in vascular inflammatory aging. Further research studies should explore the integration of prebiotics, probiotics, and/or fecal microbiota transplantation (FMT) into clinical practice and the potential of LYZ as a targeted modulator of gut microbiota [43,55,56].

While this study presents several innovative findings, they should be interpreted considering the following study limitations: (a) Lack of data validation in large, multicentric patient cohorts: The appropriate number of clinical samples determines the reliability of research results [57,58]. We used a database approach to obtain a sufficient sample size. Future studies should aim to validate these findings in larger, multicenter cohorts to ensure robustness and generalizability. (b) Limitations inherent to animal models: Although the study leverages the advantages of animal models to simulate vascular inflammatory aging in humans, caution must be exercised when extrapolating these results to humans. The inherent differences between murine and human vascular aging processes necessitate further validation in human-centric studies. (c) Lack of FMT experiments: FMT is an established method of deciphering causal relationship between gut microbiota and health and disease [59,60]. However, LYZ^−/−^ mice present with gut dysbiosis concurrent with vascular inflammatory aging. Notably, gut microbiota disruption in murine models induces vascular inflammatory aging. In this context, FMT from LYZ^−/−^ mice into either WT or GF mice can recapitulate these pathological conditions. Thus, utilization of GF mice for FMT experiments to establish causality may be redundant in this particular study design. Addressing these limitations is important to not only advance our understanding but also translate these findings into clinically actionable strategies.

**Fig. 9.**
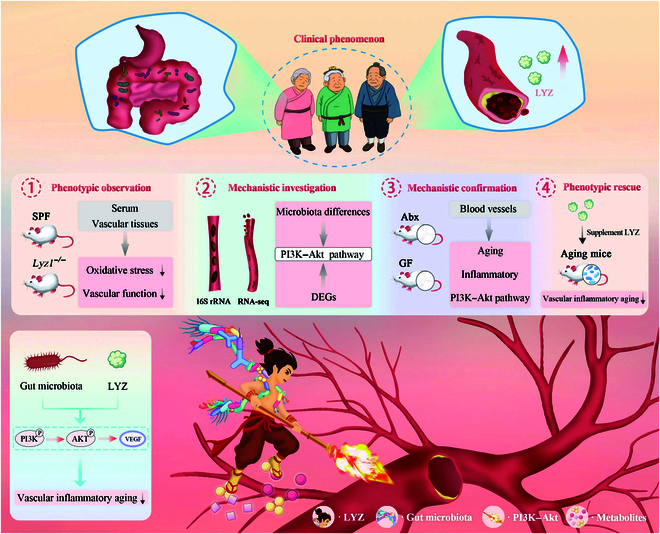
LYZ deficiency induces gut dysbiosis, which in turn promotes vascular inflammatory aging via the PI3K–Akt pathway. DEGs, differentially expressed genes; Abx, antibiotic cocktail.

In conclusion, LYZ plays a dual role in age-dependent vascular diseases, serving as both a gut-protective factor and a vascular compensatory inflammatory factor. Its deficiency induces gut dysbiosis, which in turn promotes vascular inflammatory aging via the PI3K–Akt pathway. The gut microbial imbalance resulting from LYZ deficiency may be a critical driver of vascular inflammatory aging, with mechanistic insights revealing that *Bifidobacterium* and its metabolites (e.g., butyrate) regulate the PI3K–Akt pathway to sustain vascular homeostasis. Importantly, oral administration of LYZ effectively restored gut microbiota balance and reversed vascular inflammatory aging phenotypes in our study, underscoring the necessity for future clinical strategies that prioritize gut-targeted interventions. Collectively, this study not only establishes LYZ as a novel biomarker for age-related vascular diseases and the gut microbiota–PI3K–Akt axis as a promising therapeutic target but also propels translational research forward, highlighting the potential of gut-targeted strategies for mitigating vascular inflammatory aging (Fig. 9).

## Methods

### Experimental animals and grouping

Six male specific pathogen-free (SPF) *Lyz* WT (*Lyz1^+/+^*) C57BL/6J mice aged 6 to 8 weeks and 6 *Lyz1* gene knockout (*Lyz1^−/−^*) C57BL/6J mice provided by Cyagen Biosciences Inc. were used in this study. The mice were fed with free diet for 7 d under a 12-h light/12-h dark cycle at 24 °C. Adaptive feeding was stopped after 1 week, and samples were ready to be obtained.

To verify the relationship between gut microbiota and vascular inflammatory aging, 12 male SPF WT C57BL/6J mice aged 6 to 8 weeks were randomly and equally divided into the ATBx and control (CON) groups, with 6 mice per group. Mice in the CON group were allowed free access to food and water and were housed in an SPF environment, whereas mice in the ATBx group received a combination of vancomycin (50 mg/kg, HY-B0671), imipenem (25 mg/kg, HY-B1369), neomycin (10 mg/kg, HY-B0470), and amphotericin (1 mg/kg, HY-K1008) via oral gavage at 1200 daily for 7 consecutive days. All antibiotics were procured from Shanghai Macklin Biochemical Technology Co., Ltd. (Shanghai, China). Antibiotic gavage was stopped after 7 d, after which mice were used for sampling. To avoid the impact of antibiotics on the physiological functions of mice, 6 male SPF WT C57BL/6J mice aged 6 to 8 weeks and 6 GF C57BL/6J mice were divided into CON and GF groups.

To verify the relationship between LYZ and vascular inflammatory aging, 18 male C57BL/6J mice aged 6 to 8 weeks were housed under a 12-h light/12-h dark cycle at 24 °C. The mice were divided into 3 groups, namely, natural aging group (*n* = 6), oral LYZ group (*n* = 6), and tail-vein-injected LYZ group (*n* = 6). All animals were fed with standard mouse diet for 60 weeks before intervention. The natural aging group received no treatment; in the LYZ-GAV group, LYZ (0.5 mg/kg) was administered via gastric gavage, whereas in the LYZ-IV group, LYZ (0.2 mg/kg) was administered via tail vein injection. Additionally, 6 age-matched C57BL/6J mice served as CON mice. Interventions were discontinued after 8 weeks, and samples were collected. LYZ was procured from Shanghai Macklin Biochemical Technology Co., Ltd. (Shanghai, China). GF mice were provided by the Experimental Animal Center of Huazhong Agricultural University. All operations were conducted in accordance with the Institutional Animal Care and Use Committee guidelines, and the animal protocol was approved by the Jinfeng Laboratory Experimental Animal Center (IACUC-JFLAB-2024-015).

### Tissue collection

Ileum and thoracic aorta tissues were collected and either fixed with formalin or frozen in liquid nitrogen, followed by storage at −80 °C. Fecal samples were flash-frozen in liquid nitrogen and subsequently stored at −80 °C for 16S ribosomal RNA (rRNA) sequencing analysis.

### Histopathology and immunohistochemistry staining

#### Hematoxylin–eosin, SA-β-Gal, and Alcian Blue–periodic acid–Schiff staining

Mouse ileum and thoracic aorta tissues were dissected and immediately immersed in 4% paraformaldehyde (pH 7.4) for fixation at 37 °C for 15 min. Next, the tissues were thoroughly washed with 0.1 M phosphate-buffered saline (PBS) (3 × 5 min) to remove residual fixative. Washed tissues were incubated with SA-β-Gal staining solution overnight at 37 °C in the dark. After staining, they were rinsed with PBS to terminate the reaction, and β-galactosidase-positive signals were observed under an optical microscope and recorded.

Another set of ileum and thoracic aorta tissues was immersed in 4% paraformaldehyde and fixed overnight at 4 °C. Next, gradient dehydration was performed. After xylene clearing (2 × 20 min), the dehydrated tissues were immersed in liquid paraffin at 60 °C for embedding. Subsequently, 4-μm-thick consecutive sections were prepared, attached to anti-slip slides, and baked in an oven at 60 °C for 2 h. Paraffin sections were deparaffinized with xylene and then hydrated through an ethanol gradient. Next, Alcian Blue–periodic acid–Schiff double staining was performed according to the standard protocol. Sections were counterstained with hematoxylin for 1 min, dehydrated through an ethanol gradient, and mounted with neutral balsam. Ten nonoverlapping fields of view under an optical microscope (×400 magnification) were randomly selected, and the number of goblet cells per square micrometer was calculated using the ImageJ software. The results are expressed as mean ± standard deviation.

#### Immunohistochemistry

Following the deparaffinization and rehydration of the ileum and thoracic aorta tissue sections, nonspecific antibody binding sites were blocked using a 5% bovine serum albumin blocking solution. Subsequently, the sections were incubated with the corresponding primary antibodies overnight at 4 °C. Next, the sections were washed with PBS buffer and incubated with horseradish peroxidase-conjugated secondary antibodies at 37 °C for 1 h. Diaminobenzidine was used for visualization, and hematoxylin was used for counterstaining. The primary antibodies used were LYZ (GB11345-100, Servicebio), PI3K (p85) (GB11525-100, Servicebio), PI3K (p110) (ab232997, Abcam), and Akt (GB15689-100, Servicebio).

### Flow cytometry analysis

The procedure involved collecting mouse blood in heparin coated tubes, lysing red blood cells to remove them, and then washing and resuspending the cells in PBS. The single-cell suspension was centrifuged at 500 *g* for 5 min, followed by another density gradient centrifugation at 500 *g* for 5 min at 4 °C. Subsequently, the cells were resuspended in PBS buffer and stained with F4/80 (GB113373-100, Servicebio) at 4 °C for 30 min. After incubation, the cells were washed with cold PBS and data were acquired. Data analysis was conducted using FlowJo v10.9.0.

### Enzyme-linked immunosorbent assay

Unless otherwise stated, the mice underwent fasting for 12 h before euthanasia. Mice were euthanized via cervical dislocation after isoflurane inhalation (3%), and aortic and blood samples were collected. After collecting blood from the eyeball, the serum was obtained. Levels of inflammation-related indicators (TNF-α, IL-1β, IL-10, and TGF-β), vascular function-related indicators (NO, ET-1, VEGF, and Ang II), and oxidative stress indices (SOD, MDA, catalase, glutathione peroxidase, and SA-β-Gal) were detected using enzyme-linked immunosorbent assay (Meimian, Jiangsu).

### Immunofluorescence

Paraffin-embedded thoracic aorta and ileum tissue samples were subjected to deparaffinization and rehydration, antigen retrieval, and blocking. The tissue sections were incubated overnight at 4 °C in a humidified chamber with the primary antibodies specific to the following antigens: CD31 (GB120005-100, Servicebio), H2AX (GB111841-100, Servicebio), p53 (GB90363, Abcam), p21 (ab188224, Abcam), LYZ (GB11345-100, Servicebio), and F4/80 antibody (GB113373-100, Servicebio). After washing off any unbound primary antibodies, the tissue sections were incubated with fluorescent secondary antibodies at 4 °C in the dark for 50 min in a humidified chamber. The slides were then incubated with 4′,6-diamidino-2-phenylindole (DAPI; G1012-10ML, Servicebio) and examined under a fluorescence microscope.

Image analysis was performed using ImageJ. In colocalization experiments, images from each channel were independently thresholded, with colocalization events defined as spatial overlap of at least one pixel between 2 fluorescent channels. For quantitative analysis of CD31 and H2AX colocalization, the colocalization ratio was calculated as the percentage of colocalized area relative to the total CD31-positive area. All image data remained in their original acquired state without any postprocessing modifications.

### Fluorescence in situ hybridization

Probes targeting bacterial 16S rRNA and specifically targeting *Desulfovibrio* and *Bifidobacterium* were designed to localize bacteria in the ileum. The probes and frozen tissue slides were denatured at 75 °C for 8 min and were subsequently incubated on ice for 5 min. After dehydration using an ethanol gradient, the slides were air-dried. Next, the probes were added to the slides, and hybridization was performed overnight at 37 °C in the dark. Then, the slides were washed with PBS to remove unbound probes, and immunofluorescence staining for LYZ was performed. Next, the nuclei were stained with DAPI (G1012-10ML, Servicebio). Following the addition of an anti-fade reagent (G1407-25ML, Servicebio), the stained slides were observed under a fluorescence microscope.

### Data mining and molecular docking

A total of 7 datasets were retrieved from the PubMed (https://pubmed.ncbi.nlm.nih.gov/) and Gene Expression Omnibus (https://www.ncbi.nlm.nih.gov/geo/) databases using the keyword “cardiovascular disease” (Table S1). Among them, 5 datasets (4 clinical datasets and 1 laboratory dataset) were included for statistical analysis (Fig. 1A). The expression of LYZ across different datasets was statistically analyzed using GraphPad Prism v10.2.1. Detailed methodology is provided in the Supplementary Materials.

### 16S ribosomal DNA sequencing

Microbial DNA was extracted using the E.Z.N.A. Soil DNA Kit (Omega Bio-tek, Norcross, GA, USA) according to the manufacturer’s instructions. The final DNA concentration and purification were determined using a NanoDrop 2000 ultraviolet–visible spectrophotometer (Thermo Scientific, Wilmington, USA). DNA quality was confirmed by performing 1% agarose gel electrophoresis. The V3 to V4 hypervariable region of the bacterium 16S rRNA gene was amplified using a thermocycler polymerase chain reaction system (GeneAmp 9700, ABI, USA) with primers 338F (5′-ACTCCTACGGGAGGCAGCAG-3′) and 806R (5′-GGACTACHVGGGTWTCTAAT-3′). Polymerase chain reaction products were extracted from the agarose gel, purified using an AxyPrep DNA Gel Extraction Kit (Axygen Biosciences, Union City, CA, USA), and quantified using QuantiFluor-ST (Promega, USA) according to the manufacturer’s instructions. All data analyses were conducted on the Majorbio Cloud Platform (https://cloud.majorbio.com). Detailed method is uploaded in the Supplementary Materials.

### RNA sequencing

Total RNA was extracted from renal and colonic tissues using the TRIzol reagent according to the manufacturer’s instructions (Invitrogen, USA), and genomic DNA was removed using DNase I (Takara). RNA quality was determined on 2100 Bioanalyzer (Agilent), and quantification was performed on ND-2000 (NanoDrop Technologies). Only high-quality RNA samples (1 μg of total RNA; OD260/280 = 1.8 to 2.2, OD260/230 ≥ 2.0, RNA integrity number ≥ 6.5, 28S:18S ≥ 1.0, >1 μg) were used to construct the RNA sequencing library using a TruSeq RNA Sample Prep Kit (Illumina, San Diego, USA). All data analyses were performed on the Majorbio Cloud Platform. Detailed methodology is provided in the Supplementary Materials.

### Statistical analysis

Results of the 2 groups were compared using a 2-tailed Student *t* test. Nonparametric analysis was performed using the Mann–Whitney *t* test (Wilcoxon rank-sum test). Statistical significance was determined using SPSS version 25.0 (IBM Corp., Armonk, NY, USA). A statistically significant difference was defined at *P* <0.05.

## Data Availability

Further information and requests for resources and reagents should be directed to and will be fulfilled by the lead contact, Tianhao Liu (lthlearner@126.com). The unique reagents generated in this study are available from the lead contact. All data can be provided by the corresponding author after being requested.
