# Erratum to “Gut Microbiota Modulation by Lysozyme as a Key Regulator of Vascular Inflammatory Aging”

**DOI:** 10.34133/research.1132

**Published:** 2026-02-02

**Authors:** Chenyang Zhang, Xin Zhao, Hang Zhang, Tongtong Wang, Zhenyu Zhang, Yilin Yin, Hui Wang, Xiao Tong, Yuzheng Xue, Yahong Zhou, Fenglai Yuan, Xiuwu Bian, Hong Wei, Yuan Huang, Tianhao Liu

**Affiliations:** ^1^ Institute of Integrated Traditional Chinese and Western Medicine, Affiliated Hospital of Jiangnan University, Wuxi 214122, China.; ^2^ Wuxi School of Medicine, Jiangnan University, Wuxi 214122, China.; ^3^ Department of Rehabilitation Treatment, Jiangsu Rongjun Hospital, Wuxi 214062, China.; ^4^ Department of Pathology, Army Medical University, Chongqing 400038, China.; ^5^ Yu-Yue Pathology Scientific Research Center, Jinfeng Laboratory, Chongqing 401329, China.; ^6^ Department of Pediatrics, Affiliated Hospital of Jiangnan University, Wuxi 214122, China.; ^7^ Department of Gastroenterology, Affiliated Hospital of Jiangnan University, Wuxi 214122, Jiangsu, China.; ^8^ Wuxi Hospital Affiliated to Nanjing University of Chinese Medicine, Wuxi 214071, Jiangsu, China.; ^9^ National Center for Cardiovascular Diseases, Fuwai Hospital, Chinese Academy of Medical Sciences, Peking Union Medical College, Beijing 100037, China.

In the Research Article “Gut Microbiota Modulation by Lysozyme as a Key Regulator of Vascular Inflammatory Aging,” an error occurred in panel J of Figure 5 [1]. During the final submission, the authors inadvertently uploaded a version of Figure 5 with an error in panel J and a version of Supplementary Figure 8 with an error in panel B. These figure have now been corrected in the original version and below.

**Fig. 5.**
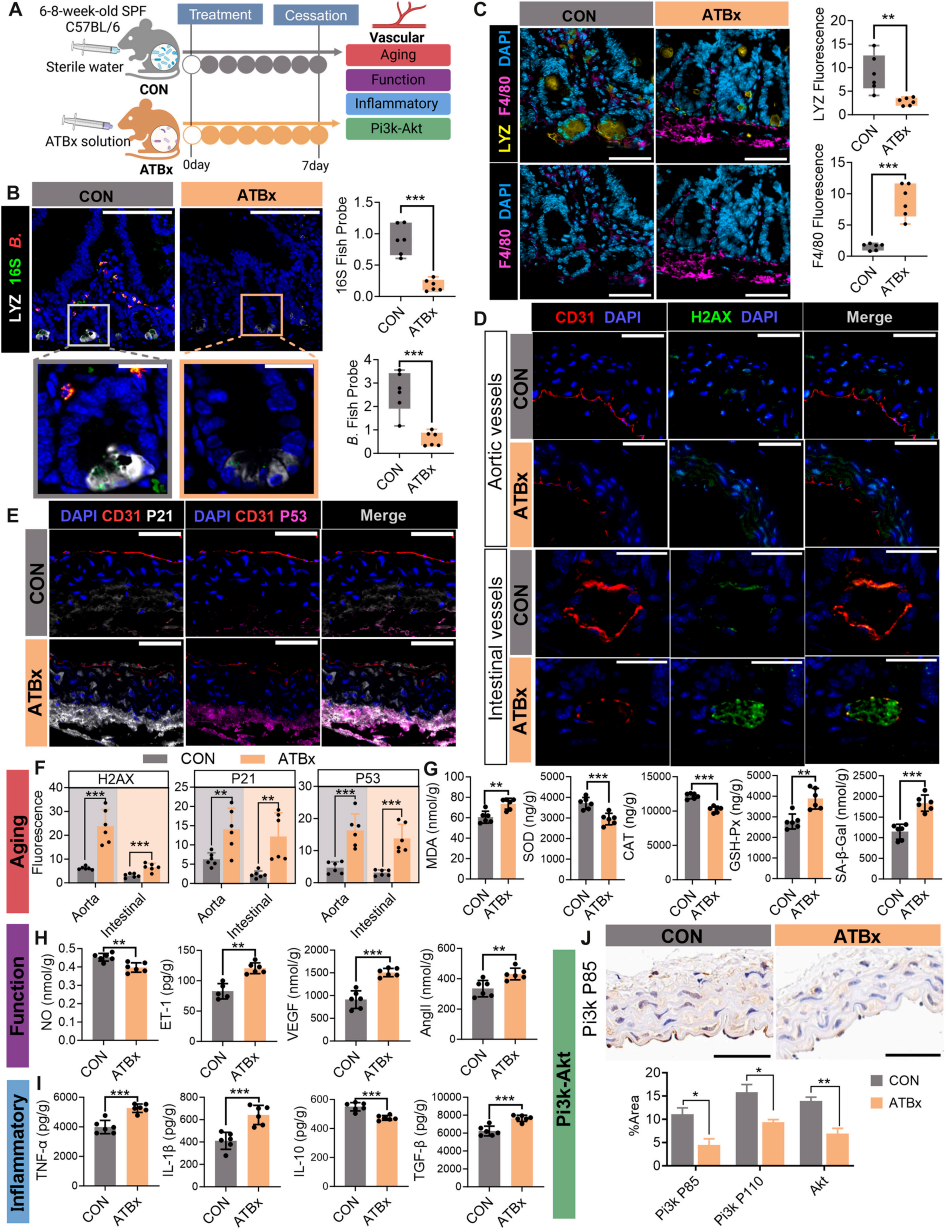
Mechanistic role of gut microbiota in Paneth cell LYZ-mediated vascular inflammation and aging revealed by ATBx mouse models. (A) Schematic of experimental design and key analytical endpoints. (B) FISH showing colocalization of LYZ and Bifidobacterium in intestinal tissues. LYZ is labeled in white, total bacteria with 16S in green, and Bifidobacterium in red. Bar graphs quantify the fluorescence intensity of LYZ colocalization with total bacteria and Bifidobacterium. Scale bars: 30 μm (×40) and 20 μm (×65). (C) Immunofluorescence colocalization of LYZ and macrophage marker F4/80. Nuclei are stained with DAPI (blue), LYZ in yellow, and F4/80 in purple. Scale bars: 30 μm (×40) and 20 μm (×65). Bar graphs compare LYZ and F4/80 fluorescence intensity between groups. (D) Immunofluorescence staining of aortic and intestinal vasculature. Nuclei are stained with DAPI (blue), CD31 (red; endothelial marker), and H2AX (green; DNA damage marker). Scale bars: 200 μm (overview) and 50 μm (magnified). (E) Immunofluorescence staining of aortic vessels. Nuclei are stained with DAPI (blue), CD31 (red), p53 (purple), and p21 (white). Scale bars: 200 μm (overview) and 50 μm (magnified). (F) Quantification of H2AX, p21, and p53 fluorescence intensity in aortic and intestinal vasculature. H2AX: DNA damage marker. (G) Alterations in vascular aging markers: MDA, SOD, CAT, GSH-Px, and SA-β-Gal. (H) Changes in vascular functional markers: NO, ET-1, VEGF, and Ang II. (I) Modulation of inflammatory markers: TNF-α, IL-1β, IL-10, and TGF-β. (J) Immunohistochemical staining and quantitative analysis of PI3K (p85), PI3K (p110), and AKT in intestinal tissues. Scale bars: 30 μm (overview) and 20 μm (magnified). Data are presented as mean ± SEM. Statistical significance: *P < 0.05; **P < 0.01; ***P < 0.001; ns, not significant. SPF, specific pathogen-free; CON, control group; ATBx, antibiotic-treated mice.

In Supplementary Figure 8, an error occurred in panel B. During the final submission, the authors inadvertently uploaded a version of Supplementary Figure 8 with an error in panel B. The figure has now been corrected in the original version and is also presented below.
