# Supplementary material for: Gut Microbiota Modulation by Lysozyme as a Key Regulator of Vascular Inflammatory Aging
Source: Research (Wash D C). 2025 May 23;8:0704. doi: 10.34133/research.0704 (PMC12099208; doi:10.34133/research.0704)
Supplement: Supplementary 1 — Supplementary Methods Table S1 Figs. S1 to S8 [file research.0704.f1.zip › RESEARCH_MT416 - clen.docx]

**Supplementary Table 1. The detailed information of bioinformatics analysis**

| **GEO** | **Disease** | **Tissue** | **Group** | ***P*** | **PMID** |
| --- | --- | --- | --- | --- | --- |
| GSE10000 | Atherosclerosis | Mice/ Aorta tissues | 6 weeks n=6  2 weeks n=6  78 weeks n=6 | 0.015 | 19139167 |
| GSE99325 | Diabetic nephropathy | Human/ Renal | Diabetic nephropathy n=18 | 0.002 | 28819298 |
| GSE145972 | Arterial stiffness | Mice/ Aortas | WT n=3  Sirt2 KO mice n =3 | *0.26* | 37377116 |
| GSE145972 | Arterial aging | Mice/ Aortas | young mice n=5  old mice n=5 | 0.26 | 32805724 |
| GSE174438 | Stroke | Mice/ Neutrophils | young mice n=4  aged mice n=4 | 0.54 | 37188941 |
| GSE174438 | Stroke | Mice/ Neutrophils | young mice n=3  aged mice n=3 | 0.032 | 37188941 |
| GSE183464 | Abdominal aortic aneurysm (AAA) | Human/ Abdominal aortic wall | AAA n=7  Control n=7 | 0.027 | 38022704 |
| GSE186844 | / | | | | |
| GSE235934 | Diabetes | Mice/ Heart | WT n=2  db/db n=3 | 0.59 | 38957358 |
| GSE262828 | Hypertension | Human/ Blood | low blood pressure n=54  elevated/mid blood pressure n=18  high blood pressure n=37 | 00012 | 38948714 |


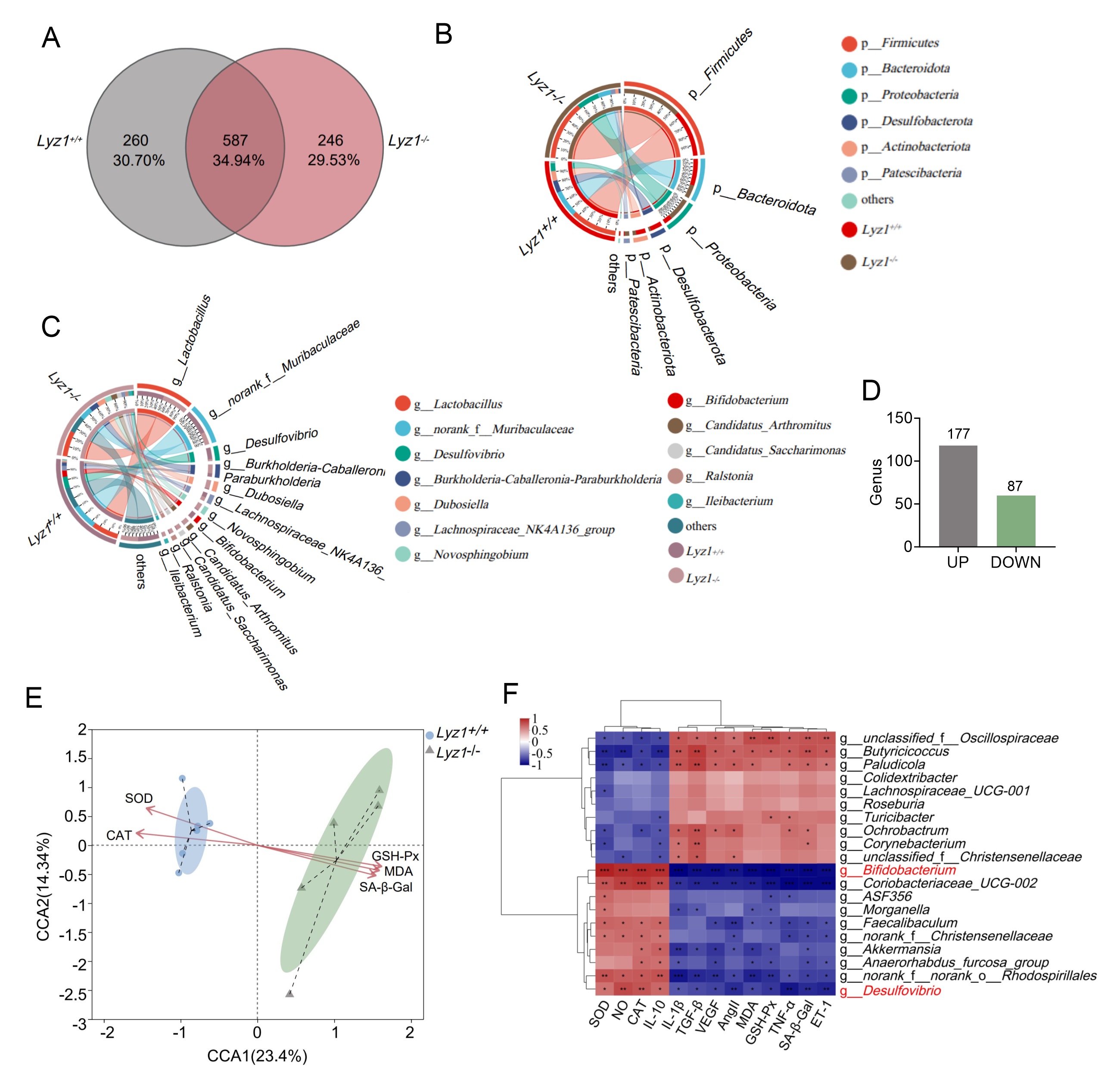


**Supplementary Figure 1.** Comparative analysis of gut microbial profiles in *Lyz1^−/−^* and *Lyz1^+/+^* mice. **(A)** Venn diagram illustrating operational taxonomic unit (OTU) overlap between gut microbiota of *Lyz1^−/−^* and *Lyz1^+/+^* mice. **(B)** Circos plot demonstrating phylum-level microbial composition differences between groups. The left semicircle displays relative abundance distribution (outer band: experimental groups; inner band: phylum classification). The right semicircle displays phylum distribution patterns across groups (outer band: bacterial phyla; inner band: group allocation). **(C)** Genus-level microbial composition analysis using Circos plot visualization. Plot configuration follows panel B description with taxonomic resolution at genus level. **(D)** Differential abundance analysis at genus level reveals 177 significantly enriched and 87 depleted bacterial taxa in *Lyz1^−/−^* mice compared to *Lyz1^+/+^* controls (*p* < 0.05 by Wilcoxon rank-sum test). **(E)** Principal coordinates analysis (PCoA) biplot showing sample clustering patterns and vascular senescence factor associations. Vector length corresponds to correlation strength (longer vectors indicate stronger associations), with acute angles (<90°) denoting positive correlations and obtuse angles (>90°) representing negative correlations. **(F)** Pearson correlation heatmap between gut microbiota abundance (x-axis: OTUs) and vascular inflammatory markers (y-axis). Color intensity reflects correlation coefficient magnitude (red: positive; blue: negative), with asterisks indicating statistical significance (**p* < 0.05; ***p* < 0.01; ****p* < 0.001). *Lyz1^+/+^*: Wild-type mice; *Lyz1^−/−^*: Lyz1-deficient mice


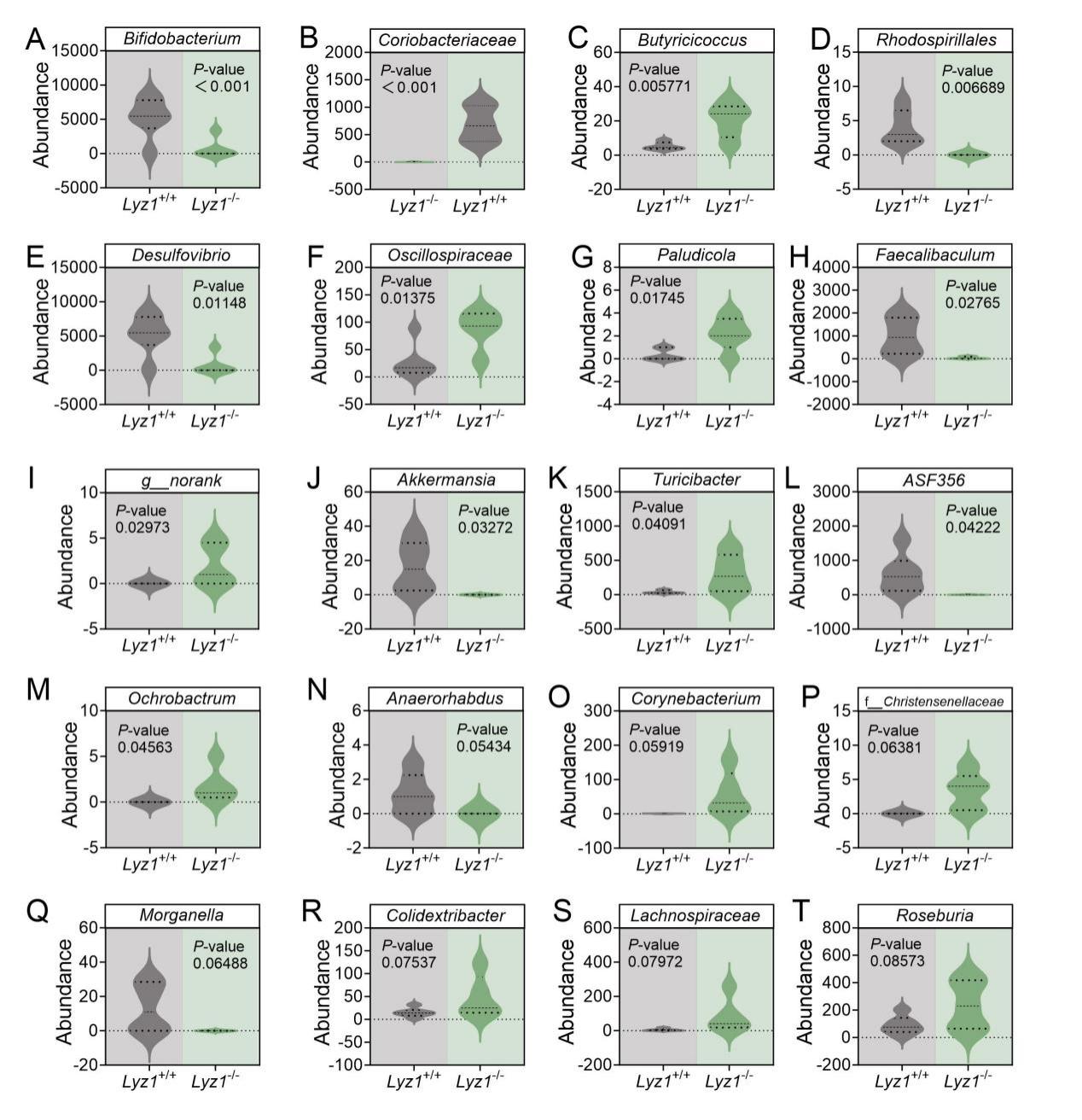


**Supplementary Figure 2. Differential abundance of the top 20 bacterial genera between *Lyz^−/−^* and *Lyz1^+/+^* mice.** Bar plots (A-T) display the relative abundance of the top 20 bacterial genera showing statistically significant differences in their abundances (*p* < 0.05 by two-tailed Student's *t*-test) between *Lyz1^−/−^* and *Lyz1^+/+^* mice at the genus level. The x-axis indicates experimental groups, and the y-axis represents the relative abundance of bacterial genera. Data are presented as mean ± SEM. *Lyz1^+/+^*: wild-type mice; *Lyz1^−/−^*: Lyz1-deficient mice


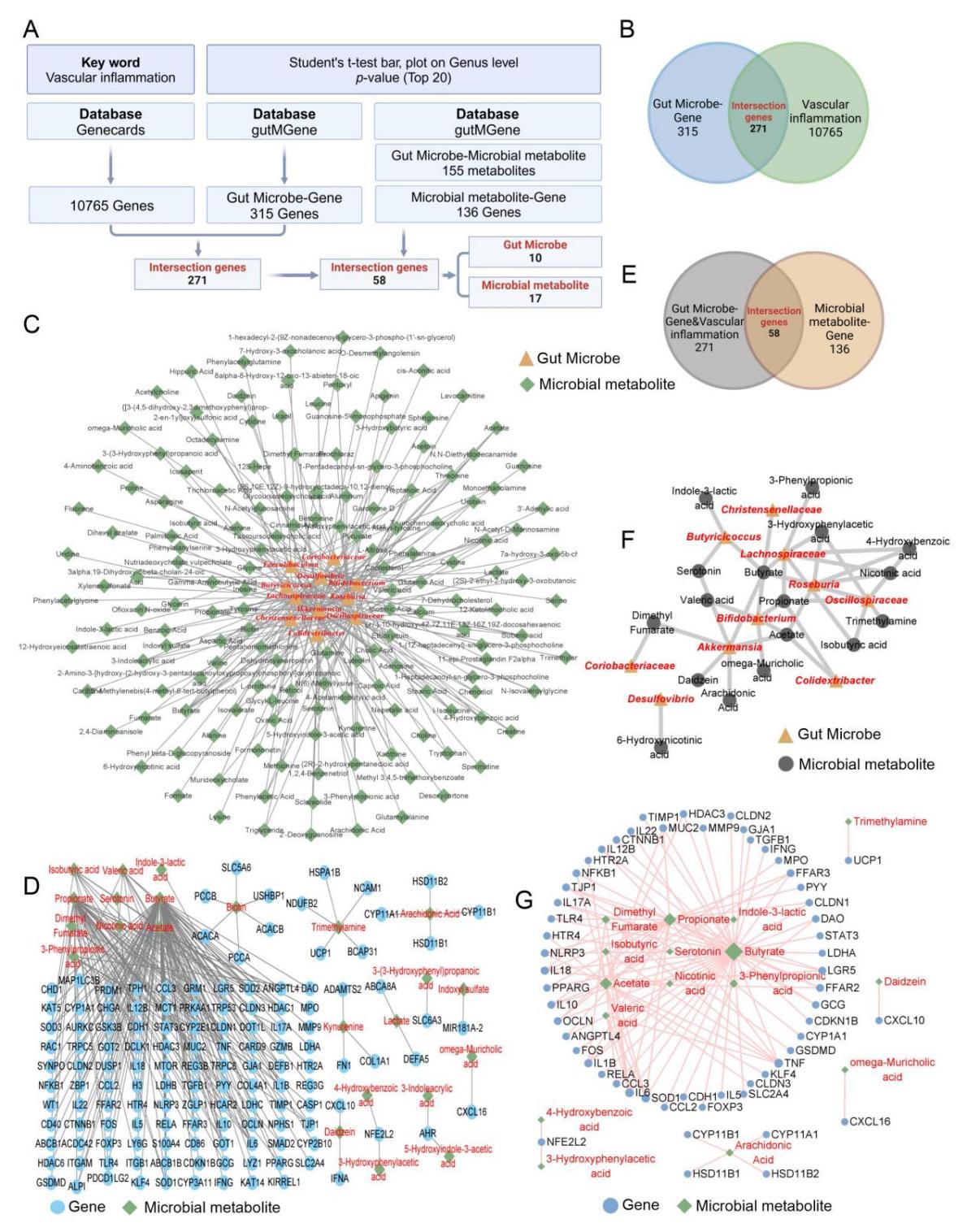


**Supplementary Figure 3. simulation of microbial metabolic environment and vascular inflammation-associated genes. (A)** Workflow and results of 16S rRNA sequencing data analysis integrated with public database mining. **(B)** Venn diagram showing 271 overlapping genes between gut microbiota-associated genes and vascular inflammation-related genes, indicating their potential involvement in vascular inflammatory pathophysiology. **(C)** Top 20 differentially abundant bacterial genera and their associated metabolites, identifying 11 bacterial genera linked to 155 distinct metabolites. **(D)** Metabolic pathway analysis revealing 136 genes associated with bacterial metabolite production. **(E)** Intersection analysis identifying 58 shared genes between vascular inflammation-related genes and microbiota metabolic genes. **(F)** Network visualization of 10 bacterial genera and 17 metabolites associated with the 58 overlapping genes. **(G)** Heatmap displaying correlations between the 17 identified metabolites and vascular inflammation-associated genes.


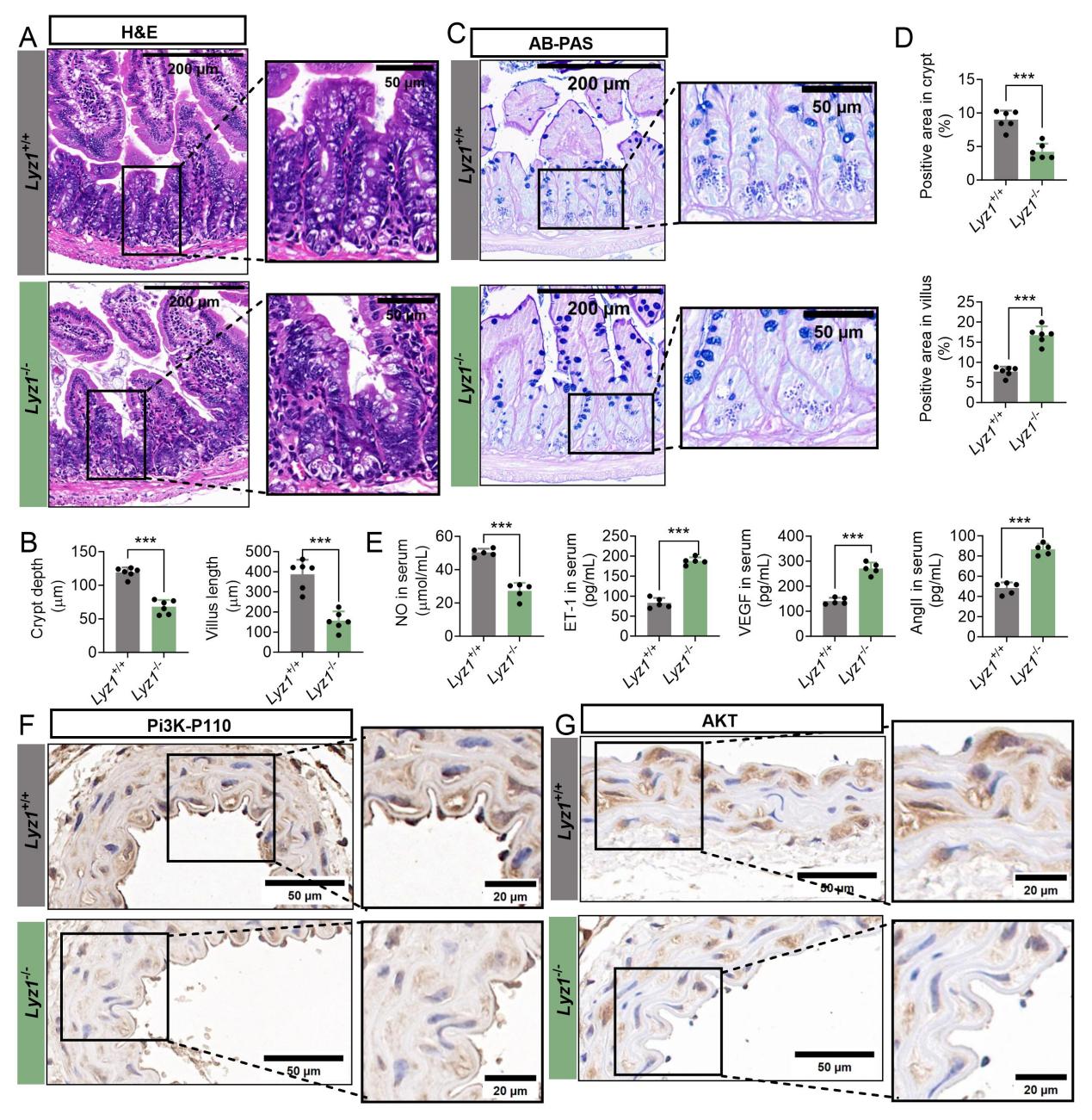


**Supplementary Figure 4. Mechanisms underlying pathological changes in vascular inflammatory aging induced in *Lyz1^-/-^* mice. (A)** Hematoxylin and eosin (HE) staining of intestinal tissues from mice. **(B)** Quantitative analysis of crypt depth and villus length in intestinal tissues. **(C)** Alcian Blue-Periodic Acid-Schiff (AB-PAS) staining of intestinal tissue sections from mice. **(D)** Quantitative analysis of mucus area in crypts and villi of intestinal tissues. **(E)** Changes in serum levels of vascular function-related markers: Nitric oxide (NO), endothelin-1 (ET-1), vascular endothelial growth factor (VEGF), and angiotensin II (Ang II). **(F)** Immunohistochemical staining of PI3k (p100) in intestinal tissues of *Lyz1-/-* mice. **(G)** Immunohistochemical staining of Akt in intestinal tissues of *Lyz1-/-* mice. PI3k: Phosphatidylinositol 3-kinase, Akt: Protein kinase B. **p* < 0.05; ***p* < 0.01; ****p* < 0.001. *Lyz1^+/+^:* Wild type mice, *Lyz1^-/-^:* Lyz1 gene knockout mice.


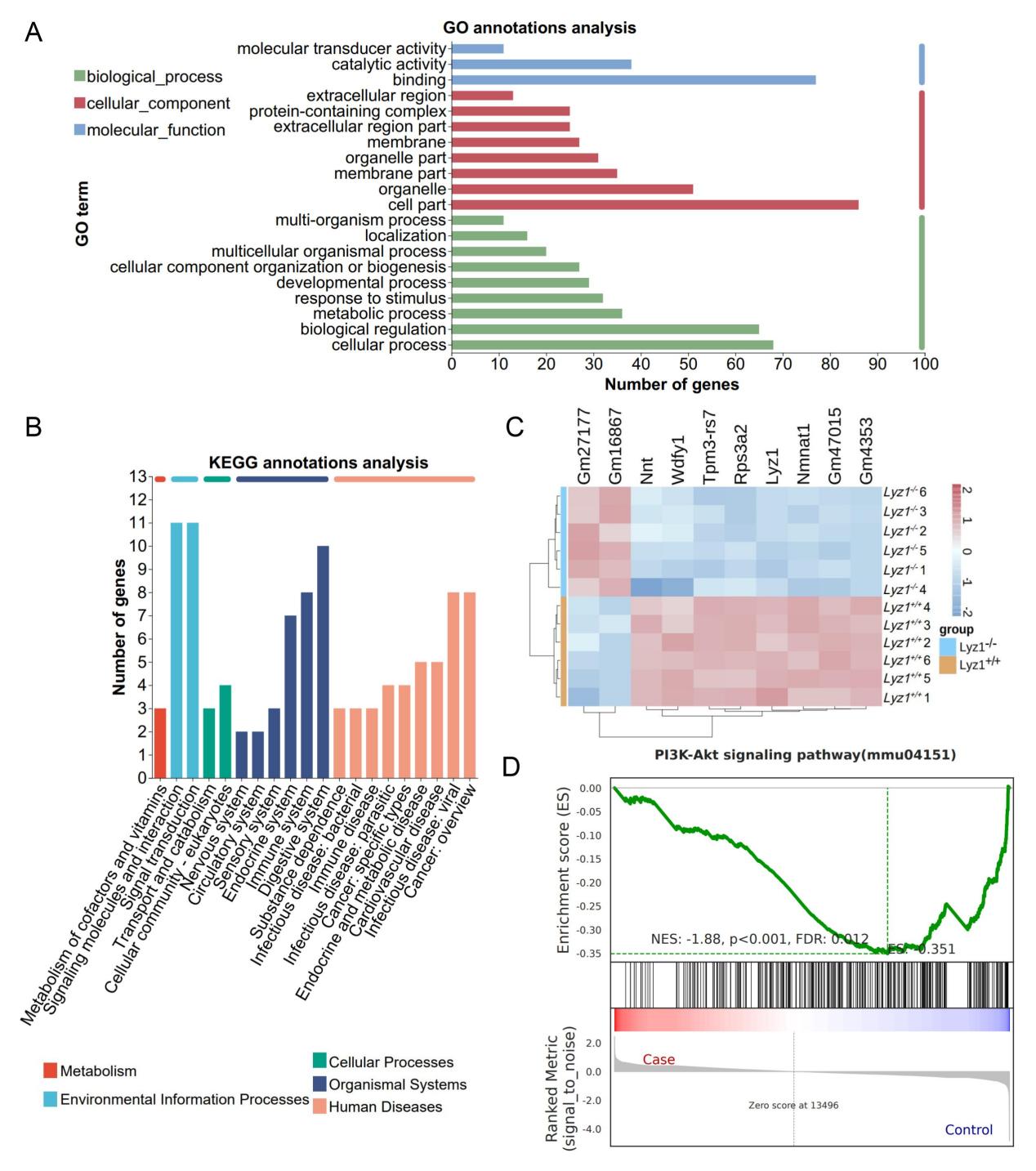


**Supplementary Figure 5. Differential gene expression and functional analysis in Lyz1^−/−^ and Lyz1^+/+^ mice. (A)** Gene Ontology (GO) enrichment analysis of differentially expressed genes. The y-axis represents GO secondary classification terms, and the x-axis indicates the number of genes annotated to each term. Color coding denotes three main GO categories: biological process (blue), cellular component (red), and molecular function (green). **(B)** KEGG annotation analysis of differentially expressed genes. The y-axis shows number of genes, and the x-axis represents KEGG's annotation results. Pathways are categorized into five functional groups: Metabolism (purple), Environmental Information Processing (orange), Cellular Processes (green), Organismal Systems (blue), and Human Diseases (red). **(C)** Heatmap visualization of the top 10 differentially expressed genes between *Lyz1^−/−^* and *Lyz1^+/+^* mice. Color intensity represents gene expression levels, with red indicating high expression and blue indicating low expression. **(D)** Gene Set Enrichment Analysis (GSEA) of the PI3K-Akt signaling pathway. Enrichment score (ES) = -0.351. The main plot shows the running enrichment score, with genes to the right of the peak representing core enriched genes. Vertical lines below the plot indicate the position of genes in the ranked list. The bottom panel displays the signal to noise metric distribution across the ranked gene list. Lyz1^+/+^: wild-type mice; Lyz1^−/−^: Lyz1-deficient mice.


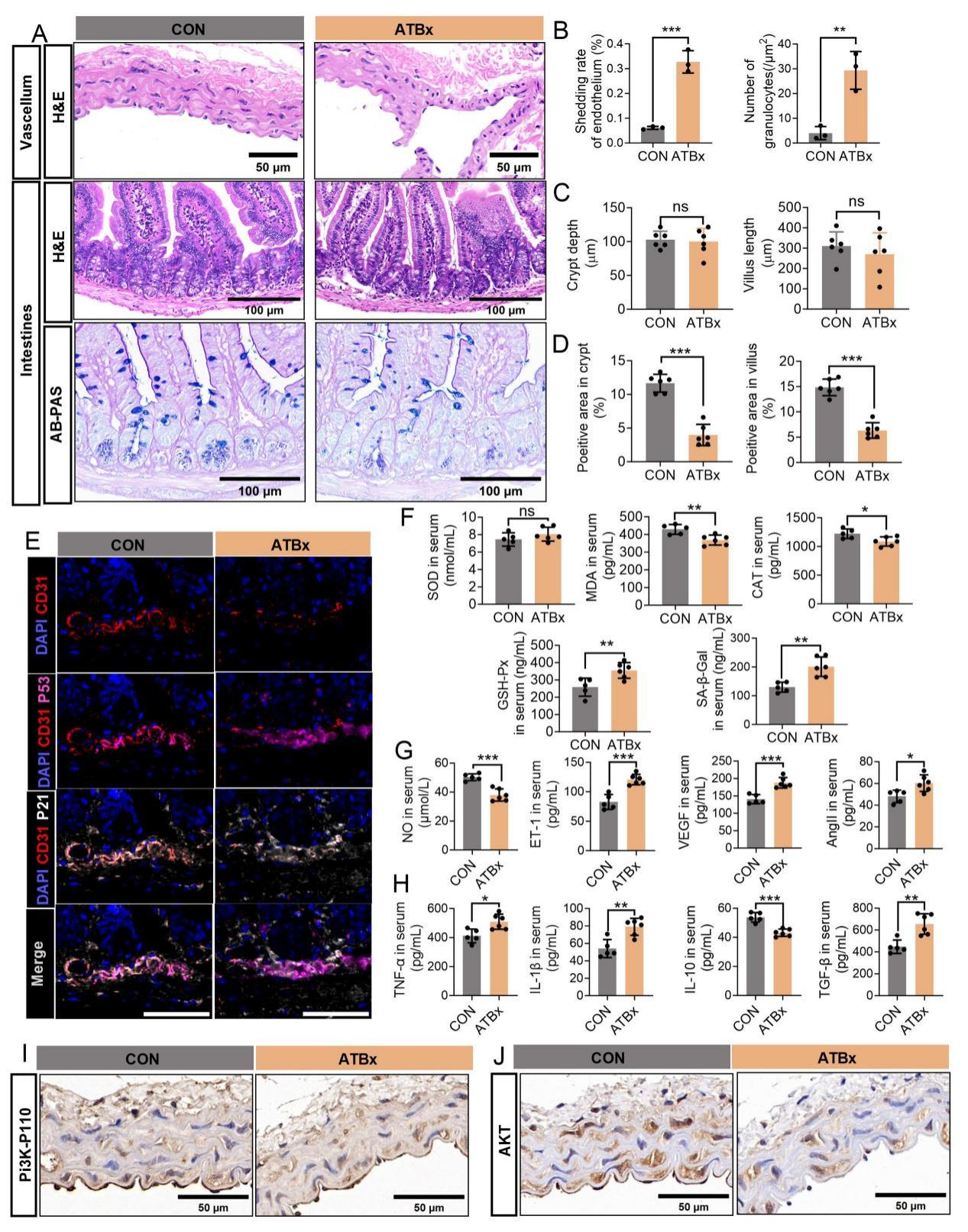


**Supplementary Figure 6. Mechanisms underlying pathological changes in vascular inflammatory aging induced in ATBx mice. (A)** Hematoxylin and eosin (HE) staining of aortic vessels and intestinal tissues, and Alcian Blue-Periodic Acid-Schiff (AB-PAS) staining of intestinal tissues. **(B)** Quantitative analysis of endothelial cell detachment rate and granulocyte count in aortic vessels. **(C)** Quantitative analysis of crypt depth and villus length in intestinal tissues. **(D)** Quantitative analysis of mucus area in crypts and villi of intestinal tissues**. (E)** Immunofluorescence staining of intestinal vasculature. Nuclei were stained with DAPI (blue), CD31 (red), P53 (purple), and P21 (white). Scale bars: 200 μm (overview), 50 μm (magnified view). **(F)** Changes in serum levels of aging-related markers: malondialdehyde (MDA), superoxide dismutase (SOD), catalase (CAT), glutathione peroxidase (GSH-Px), and β-galactosidase (SA-β-gal). **(G)** Changes in serum levels of vascular function-related markers: nitric oxide (NO), endothelin-1 (ET-1), vascular endothelial growth factor (VEGF), and angiotensin II (Ang II). (H) Changes in serum levels of inflammatory markers: tumor necrosis factor-α (TNF-α), interleukin-1β (IL-1β), interleukin-10 (IL-10), and transforming growth factor-β (TGF-β). **(I)** Immunohistochemical staining of PI3k (p100) in intestinal tissues of ATBx mice. **(J)** Immunohistochemical staining of Akt in intestinal tissues of ATBx mice. PI3k: Phosphatidylinositol 3-kinase; Akt: Protein kinase B. **p* < 0.05; ***p* < 0.01; ****p* < 0.001; ns, not significant. CON: Control group; ATBx: Antibiotics-treated mice.


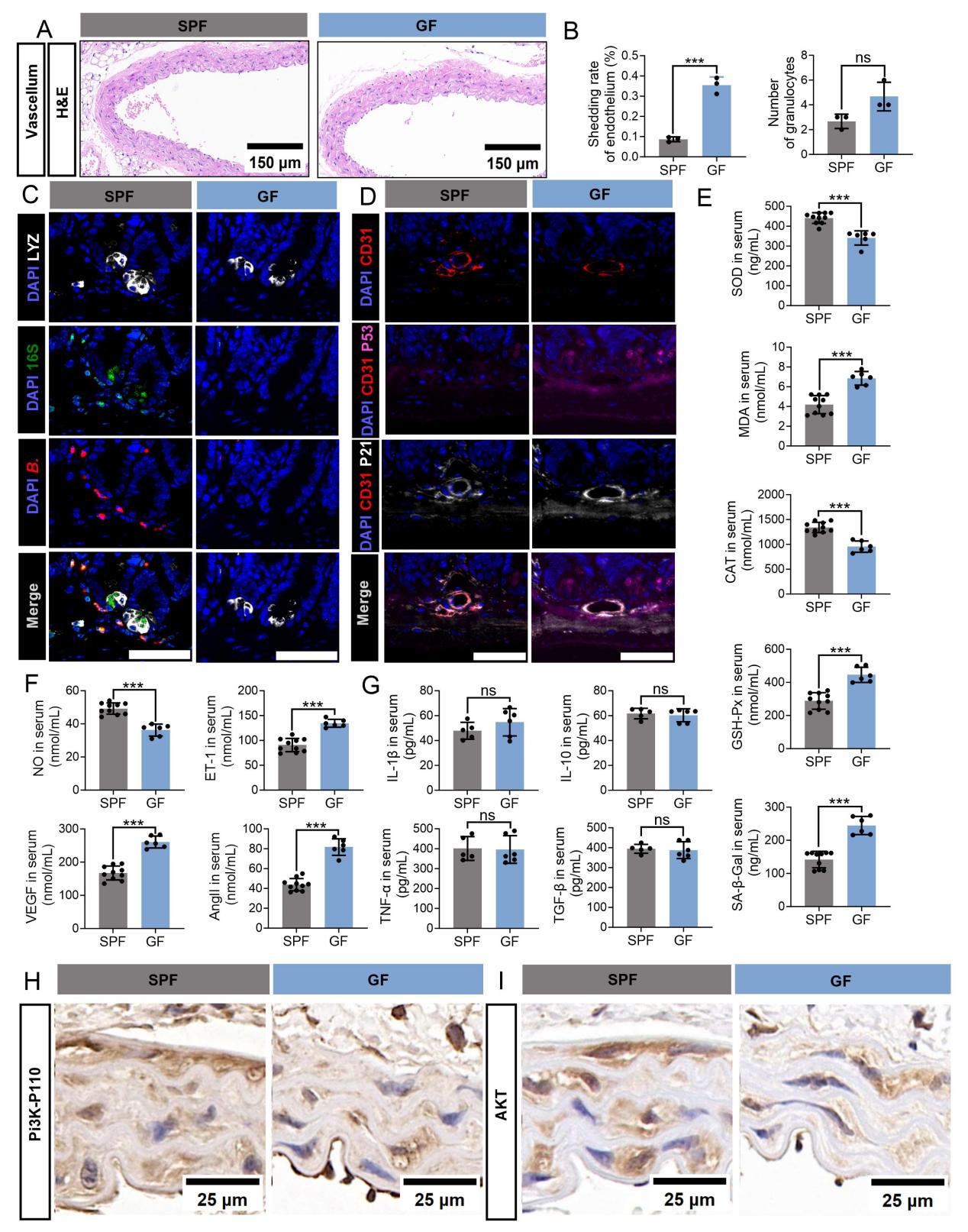


**Supplementary Figure 7. Mechanisms underlying pathological changes in vascular inflammatory aging induced in germ-free (GF) mice.** **(A)** Hematoxylin and eosin (HE) staining of aortic vessels. **(B)** Quantitative analysis of endothelial cell detachment rate and granulocyte count in aortic vessels. **(C)** Fluorescence in situ hybridization (FISH) showing co-localization and fluorescence intensity quantification of LYZ and Bifidobacterium in the intestinal tissues of both groups. LYZ is shown in white, total bacteria labeled for 16S rDNA is shown in green, and Bifidobacterium is shown in red. **(D)** Immunofluorescence staining of intestinal vasculature. Nuclei were stained with DAPI (blue), CD31 (red), P53 (purple), and P21 (white). Scale bars: 200 μm (overview), 50 μm (magnified view). **(E)** Changes in serum levels of aging-related markers: malondialdehyde (MDA), superoxide dismutase (SOD), catalase (CAT), glutathione peroxidase (GSH-Px), and β-galactosidase (SA-β-gal). **(F)** Changes in serum levels of vascular function-related markers: nitric oxide (NO), endothelin-1 (ET-1), vascular endothelial growth factor (VEGF), and angiotensin II (Ang II). **(G)** Changes in serum levels of inflammatory markers: tumor necrosis factor-α (TNF-α), interleukin-1β (IL-1β), interleukin-10 (IL-10), and transforming growth factor-β (TGF-β). (H) Immunohistochemical staining of Pi3k (p100) in intestinal tissues of GF mice. **(I)** Immunohistochemical staining of Akt in intestinal tissues of GF mice. PI3k: Phosphatidylinositol 3-kinase; Akt: Protein kinase B. **p* < 0.05; ***p* < 0.01; ****p* < 0.001; ns, not significant. CON: Control group; GF: Germ-free mice.


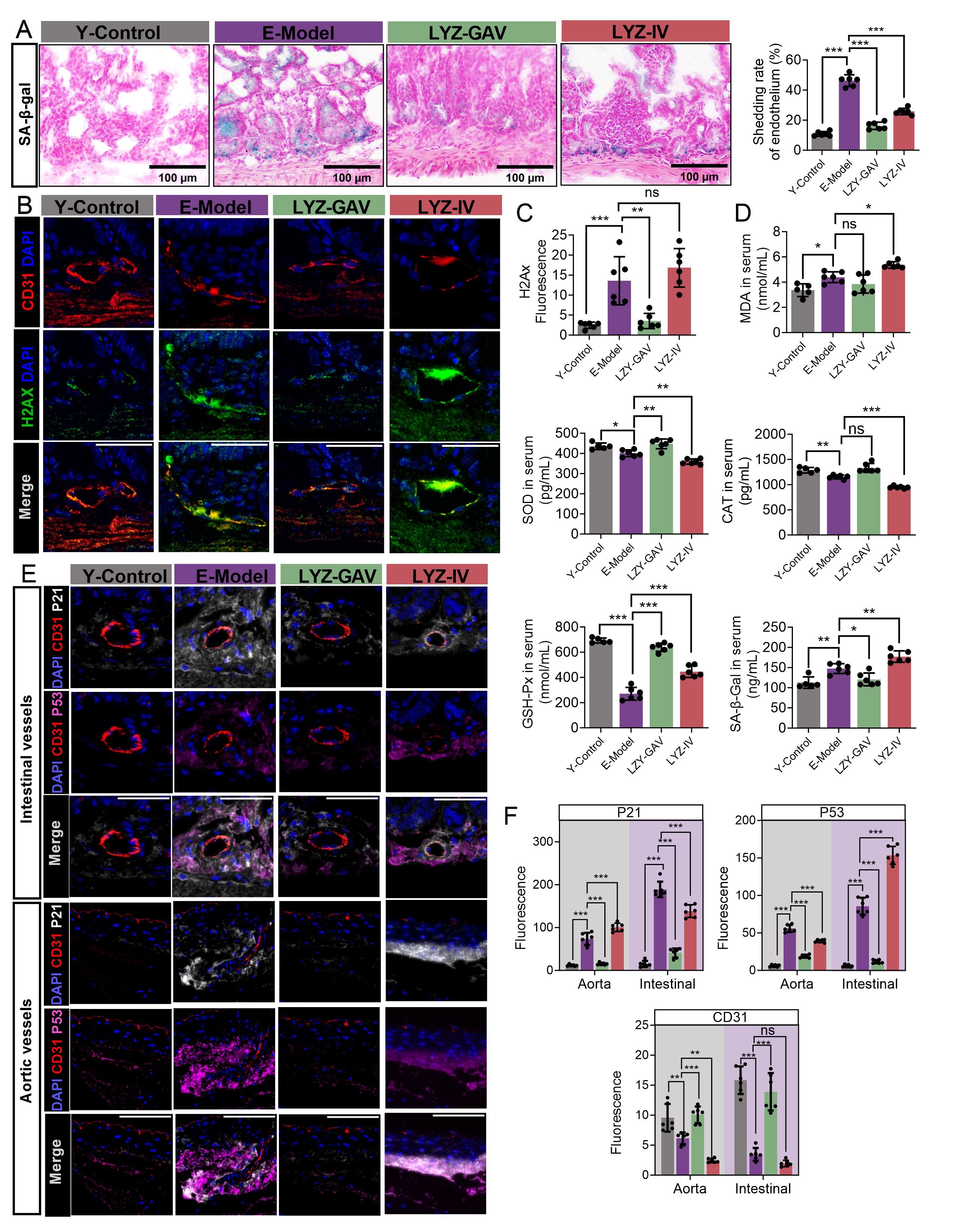


**Supplementary Figure 8. Mechanisms underlying the alleviation of vascular inflammatory aging by oral LYZ administration. (A)** Expression and quantitative analysis of SA-β-gal in intestinal tissues of mice from each group. **(B)** Immunofluorescence staining of intestinal vasculature. Nuclei were stained with DAPI (blue), CD31 (red), and H2AX (green). Scale bars: 200 μm (overview), 50 μm (magnified view). H2AX: DNA damage marker. **(C)** Quantitative analysis of H2AX fluorescence intensity in intestinal vasculature. **(D)** Changes in serum levels of aging-related markers: malondialdehyde (MDA), superoxide dismutase (SOD), catalase (CAT), glutathione peroxidase (GSH-Px), and β-galactosidase (SA-β-gal). **(E)** Immunofluorescence staining of intestinal vasculature and aortic tissues. Nuclei were stained with DAPI (blue), CD31 (red), P53 (purple), and P21 (white). Scale bars: 200 μm (overview), 50 μm (magnified view). **(F)** Quantitative analysis of fluorescence intensity for P21, P53, and CD31 in intestinal vasculature and aortic tissues. **p* < 0.05; ***p* < 0.01; ****p* < 0.001; ns, not significant. Y-Control: Young control group; E-Model: Elderly model group; LYZ-GAV: LYZ gavage group; LYZ-IV: LYZ intravenous injection group.
