# Supplementary material for: Gut Microbiota Modulation by Lysozyme as a Key Regulator of Vascular Inflammatory Aging
Source: Research (Wash D C). 2025 May 23;8:0704. doi: 10.34133/research.0704 (PMC12099208; doi:10.34133/research.0704)
Supplement: Supplementary 1 — Supplementary Methods Table S1 Figs. S1 to S8 [file research.0704.f1.zip › Supplementary_Information415.docx]

**Supplementary Information**

***Supplementary Methods***

The top 20 bacteria at the Genus level were input into the gutMGene database (http://bio-annotation.cn/gutmgene/home.dhtml) for in-depth mining. The corresponding metabolically active bacteria, metabolites, gut microbiota-related genes and metabolite-related genes were identified. We summarized the genes related to vascular inflammatory diseases using the keyword 'Vascular Inflammation' through the GeneCards database (https://www.genecards.org/). Through docking-based identification of microbiota-associated genes implicated in vascular inflammatory pathologies, the gutMGene in silico simulation system systematically screened gut microbial metabolites and metabolite-linked genes, thereby elucidating the core interaction network underlying the gut microbiota-gene-disease axis.

***Molecular Docking***

The structural file of Lysozyme (PDB ID: 5LSH) was downloaded from the PDB database (https://www1.rcsb.org/). The molecular structure of the peptidoglycan, a small molecule, was drawn using InDraw5.0, and then optimized by the MMFF94 force field of the OpenBabel3.1.1 software to obtain the optimal structure with the lowest energy. The protein was hydrogenated using AutoDock Tools1.5.6, and the peptidoglycan small molecule was hydrogenated and its rotatable bonds were determined. Then both were saved as pdbqt files. The parameters of the molecular docking range were set in the Grid module, with the Center (X, Y, Z) = (5.0, 15.0, 12.6) and Size (X×Y×Z) = (44×44×49). The docking mode was set as semi-flexible docking, the docking accuracy (exhaustiveness) was set to 25, and the Lamarckian genetic algorithm was adopted. The Auto Dock Vina 1.2.0 software was run for molecular docking to obtain the docking binding free energy and the docking result files.

***FISH probe sequences***

Universal primers targeting bacterial 16S rDNA were selected from the EUB338 commercial kit (sequence: 5'-GCTGCCTCCCGTAGGAGT-3'). Complete genome sequences of Desulfovibrio and Bifidobacterium were retrieved from NCBI (https://www.ncbi.nlm.nih.gov). Genomic annotation was performed using Prokka (v1.11), followed by identification of all core genes with Roary (v3.6.1) under a 95% protein amino acid similarity threshold. Consensus core gene sequences were aligned against the NCBI nucleotide database (nt) using BLAST (v2.2.24+). Sequences exhibiting >97% similarity to non-target species were eliminated, retaining species-specific consensus sequences. Species-specific primers were designed targeting conserved single-copy core genes: *Desulfovibrio*-specific primer: 5'-CGCAGGCGATGCA(G/C)GGGTC-3'. *Bifidobacterium*-specific primer: 5'-AGAAGTCCAAGACTTTGGCCCTGA-3'. All FISH probes were synthesized by Wuhan Servicebio Technology Co., Ltd

**16S rDNA sequencing**

16S rDNA sequencing was outsourced to the Majorbio Bio-Pharm Technology Co. Ltd. They pooled purified amplicons in an equimolar ratio and sequenced them (2 × 300) on an Illumina MiSeq platform (Illumina, San Diego, USA) (Shanghai, China). The operational taxonomic units (OTUs) were clustered with a 97% similarity criterion using UPARSE (version 7.1; http://drive5.com/uparse/) with a unique 'greedy' approach that performs chimera filtering and OTU clustering simultaneously after raw fastq files were quality-filtered and merged. Finally, the taxonomy of each 16S rRNA gene sequence was compared to the Silva (SSU123) 16S rRNA database using the RDP Classifier algorithm (http://rdp.cme.msu.edu/) with a confidence threshold of 70%.

All data analyses were conducted on the Majorbio Cloud Platform (https://cloud.majorbio.com), as detailed below: The Alpha diversity indices, including Chao 1 and Shannon, were calculated using the mothur software (http://www.mothur.org/wiki/Calculators). Principal Coordinate Analysis (PCoA) based on the Bray-Curtis distance algorithm was employed to examine the similarities in microbial community structures among samples. LEfSe analysis (Linear Discriminant Analysis Effect Size) (http://huttenhower.sph.harvard.edu/LEfSe) (LDA > 2, *p* < 0.05) was used to identify bacterial taxa with significantly different abundances from the phylum to genus levels across different groups. Circos plots illustrating species abundance were generated at both the phylum and genus levels. In the phylum- and genus-level analyses, taxa with a relative abundance below 3% were grouped as "others" . Canonical Correspondence Analysis (CCA) was performed at the OTU level to investigate the correlations between microbial community structure and SOD, CAT, GSH-Px, and SA-β-Gal levels. At the genus level, Pearson correlation analysis was performed to assess the relationships between microbial communities and SOD, CAT, GSH-Px, SA-β-Gal, NO, ET-1, VEGF, AngII, TNF-α, IL-1β, IL-10, and TGF-β levels. Both row and column clustering were conducted using the Average method.

**RNA-sequencing**

Following quantification using TBS380, the paired-end RNA-seq sequencing library was sequenced on the Illumina HiSeq xten/NovaSeq 6000 sequencer (2 × 150 bp read length). The transcripts per million reads method was used to calculate the expression level of each transcript and detect differentially expressed genes (DEGs) between two groups. Gene abundances were quantified using RSEM (http://deweylab.biostat.wisc.edu/rsem/). Functional enrichment analyses, including the Kyoto encyclopedia of genes and genomes (KEGG), were performed to determine which DEGs were significantly enriched in metabolic pathways. KEGG pathway analyses were conducted using Goatools (https://github.com/tanghaibao/Goatools) and KOBAS (http://kobas.cbi.pku.edu.cn/home.do). All data analyses were performed on the Majorbio Cloud Platform (https://cloud.majorbio.com). First, a volcano plot of differentially expressed genes was generated, with a screening threshold set at a fold change of |log2FC| > 2, and the genes Col6a6, Col6a5, Col4a5, and Col1a2 were specifically highlighted. Second, a heatmap illustrating the correlation between microbial communities at the genus level and differentially expressed genes was created, using Spearman correlation coefficients for calculation, with both row and column clustering performed using the Average method. Additionally, functional annotation of differentially expressed genes was conducted using the GO database, categorizing them according to their involvement in biological processes (BP), cellular components (CC), and molecular functions (MF). Meanwhile, pathway annotation of differentially expressed genes was performed based on the KEGG database, classifying them according to their participation in metabolic pathways or biological functions, thereby comprehensively elucidating the functional roles of the genes and their correlations with microbial communities. Using the OE Cloud Platform (https://cloud.oebiotech.com/#/home), a heatmap was generated for the top 10 differentially expressed genes, with data processing following the platform's default settings. Additionally, Gene Set Enrichment Analysis (GSEA) was performed on all genes, with the minimum and maximum gene set sizes set to 15 and 500, respectively. The KEGG database was selected as the gene set database, and the signal-to-noise method was used for calculation.
