# Supplementary material for: Erratum to “Gut Microbiota Modulation by Lysozyme as a Key Regulator of Vascular Inflammatory Aging”
Source: Research (Wash D C). 2026 Feb 2;9:1132. doi: 10.34133/research.1132 (PMC12862132; doi:10.34133/research.1132)
Supplement: Supplementary 1 — Fig. S8 [file research.1132.f1.zip › sm.docx]

**
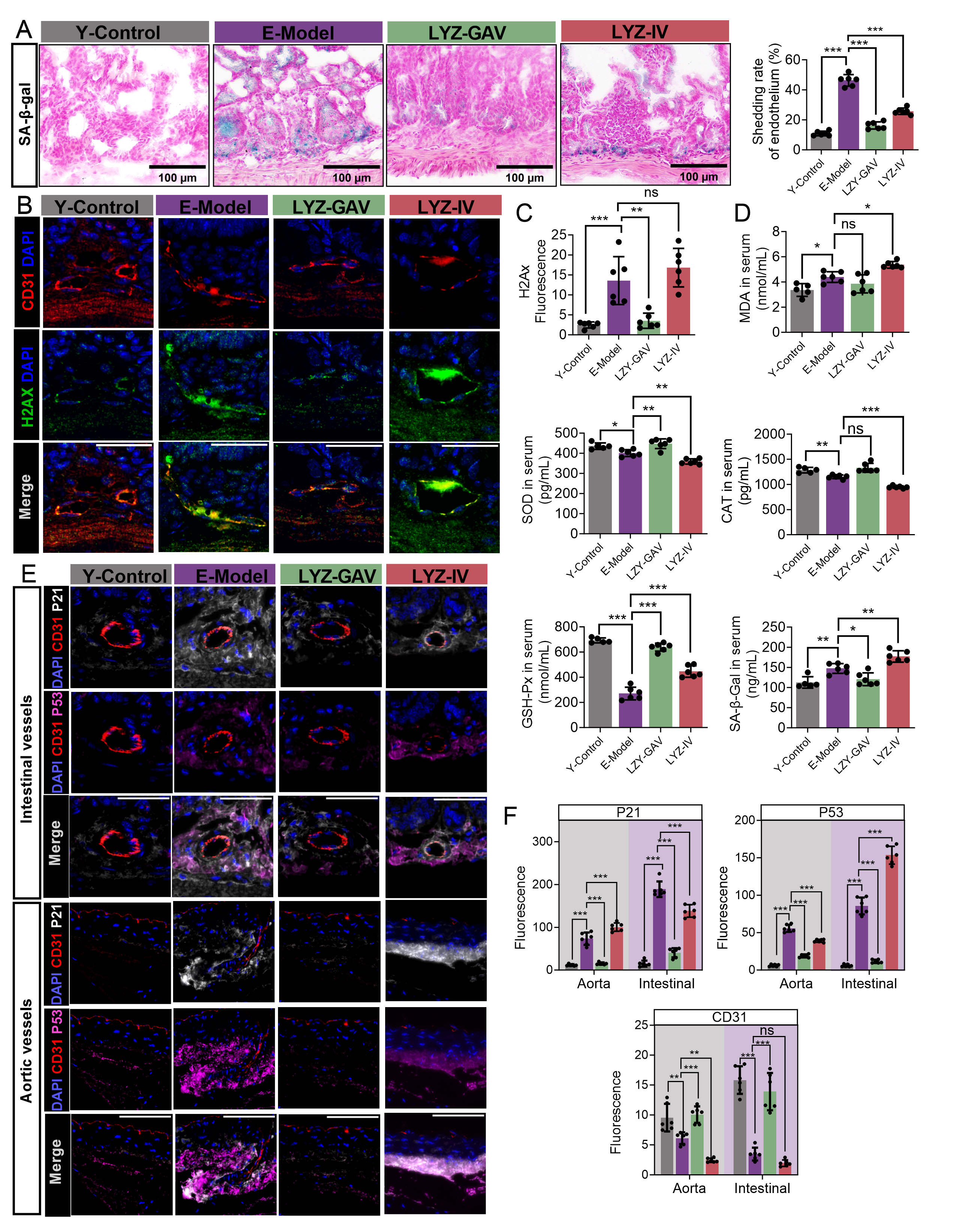
**

**Supplementary Figure 8. Mechanisms underlying the alleviation of vascular inflammatory aging by oral LYZ administration. (A)** Expression and quantitative analysis of SA-β-gal in intestinal tissues of mice from each group. **(B)** Immunofluorescence staining of intestinal vasculature. Nuclei were stained with DAPI (blue), CD31 (red), and H2AX (green). Scale bars: 200 μm (overview), 50 μm (magnified view). H2AX: DNA damage marker. **(C)** Quantitative analysis of H2AX fluorescence intensity in intestinal vasculature. **(D)** Changes in serum levels of aging-related markers: malondialdehyde (MDA), superoxide dismutase (SOD), catalase (CAT), glutathione peroxidase (GSH-Px), and β-galactosidase (SA-β-gal). **(E)** Immunofluorescence staining of intestinal vasculature and aortic tissues. Nuclei were stained with DAPI (blue), CD31 (red), P53 (purple), and P21 (white). Scale bars: 200 μm (overview), 50 μm (magnified view). **(F)** Quantitative analysis of fluorescence intensity for P21, P53, and CD31 in intestinal vasculature and aortic tissues. **P* < 0.05; ***P* < 0.01; ****P* < 0.001; ns, not significant. Y-Control: Young control group; E-Model: Elderly model group; LYZ-GAV: LYZ gavage group; LYZ-IV: LYZ intravenous injection group.
