# Supplementary figures and images for: Erratum to “Gut Microbiota Modulation by Lysozyme as a Key Regulator of Vascular Inflammatory Aging”
Source: Research (Wash D C). 2026 Feb 2;9:1132. doi: 10.34133/research.1132 (PMC12862132; doi:10.34133/research.1132)

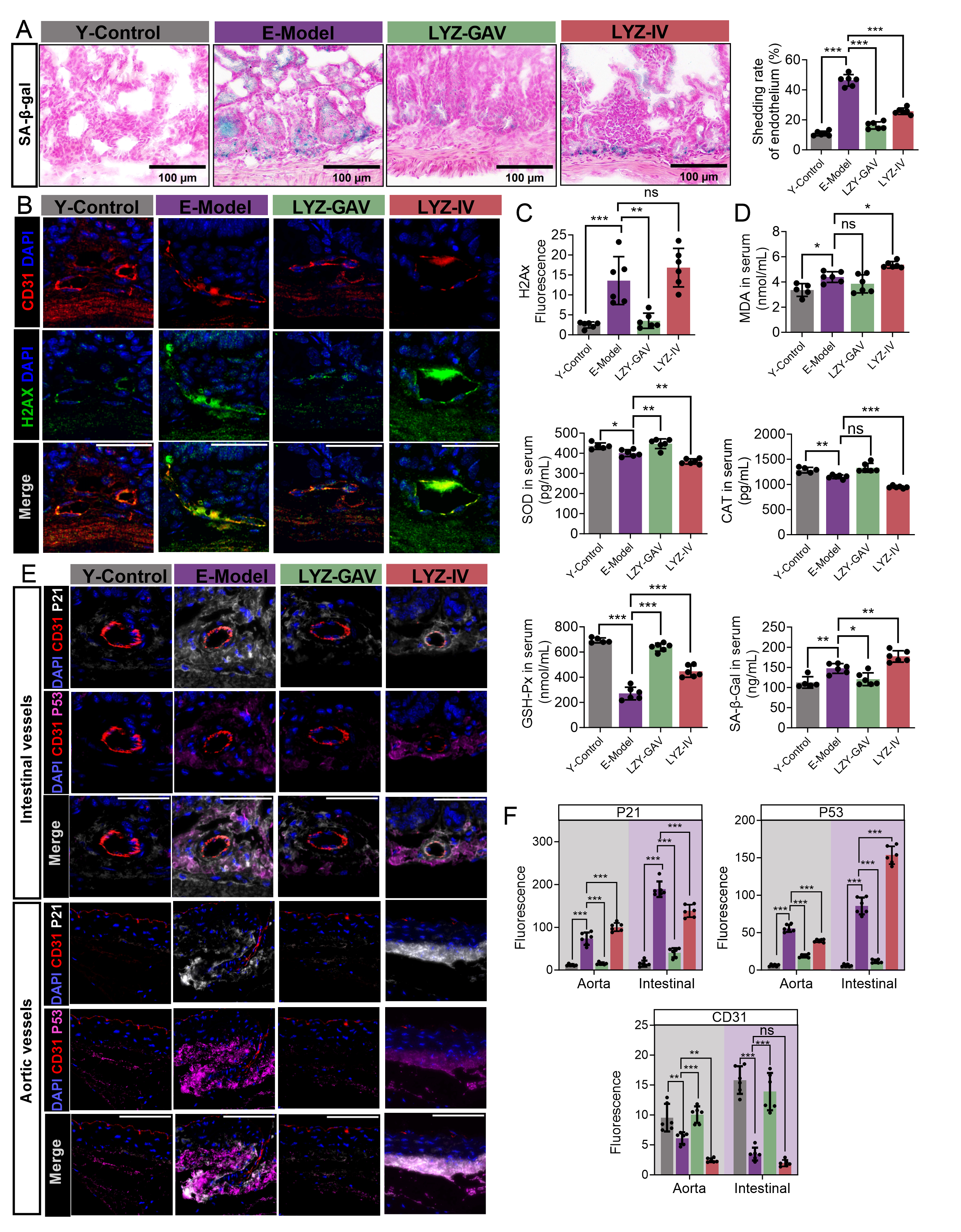

Supplement: Supplementary 1 — Fig. S8 [file research.1132.f1.zip › Supplymentray Fig8..tif]
